# Identification of Distinct Immune Cell Subsets Associated With Asymptomatic Infection, Disease Severity, and Viral Persistence in COVID-19 Patients

**DOI:** 10.3389/fimmu.2022.812514

**Published:** 2022-02-22

**Authors:** Xiaorui Wang, Han Bai, Junpeng Ma, Hongyu Qin, Qiqi Zeng, Fang Hu, Tingting Jiang, Weikang Mao, Yang Zhao, Xiaobei Chen, Xin Qi, Mengyang Li, Jiao Xu, Jingcan Hao, Yankui Wang, Xi Ding, Yuanrui Liu, Tianlong Huang, Chao Fang, Changli Ge, Dong Li, Ke Hu, Xianwen Ren, Baojun Zhang, Binghong Zhang, Bingyin Shi, Chengsheng Zhang

**Affiliations:** ^1^ Precision Medicine Center, The First Affiliated Hospital of Xi’an Jiaotong University, Xi’an, China; ^2^ The MED-X Institute, The First Affiliated Hospital of Xi’an Jiaotong University, Xi’an, China; ^3^ LC-Bio Technologies, Co., Ltd., Hangzhou, China; ^4^ Department of Respiratory and Critical Care Medicine, Renmin Hospital of Wuhan University, Wuhan, China; ^5^ Department of Infectious Diseases, The Renmin Hospital of Wuhan University, Wuhan, China; ^6^ Department of Endocrinology, The First Affiliated Hospital of Xi’an Jiaotong University, Xi’an, China; ^7^ Cancer Center, The First Affiliated Hospital of Xi’an Jiaotong University, Xi’an, China; ^8^ Dialysis Center, The Renmin Hospital of Wuhan University, Wuhan, China; ^9^ Department of Clinical Laboratory, The Renmin Hospital of Wuhan University, Wuhan, China; ^10^ Biomedical Pioneering Innovation Center (BIOPIC), School of Life Sciences, Peking-Tsinghua Center for Life Sciences, Academy for Advanced Interdisciplinary Studies, Beijing Advanced Innovation Center for Genomics (ICG), Peking University, Beijing, China; ^11^ Department of Pathogenic Microbiology and Immunology, School of Basic Medical Sciences, Xi’an Jiaotong University, Xi’an, China; ^12^ The Renmin Hospital of Wuhan University, Wuhan, China; ^13^ The Jackson Laboratory for Genomic Medicine, Farmington, CT, United States

**Keywords:** SARS-CoV-2, COVID-19, asymptomatic infection, disease severity, viral persistence, singlecell RNA sequencing (scRNA-seq)

## Abstract

The cell-mediated protective and pathogenic immune responses to SARS-CoV-2 infection remain largely elusive. Here we identified 76 distinct cell subsets in the PBMC samples that were associated with various clinical presentations of COVID-19 using scRNA-seq technology coupled with a deep and comprehensive analysis of unique cell surface markers and differentially expressed genes. We revealed that (TRAV1-2^+^CD8^+^)MAIT cells and (NCAM1^hi^CD160^+^)NK cells significantly enriched in the asymptomatic subjects whereas (LAG3^+^CD160^+^CD8^+^)NKT cells increased in the symptomatic patients. We also observed that (CD68^-^CSF1R^-^IL1B^hi^CD14^+^)classical monocytes were positively correlated with the disease severity. Moreover, (CD33^-^HLA-DMA^-^CD14^+^)classical monocytes and (CLEC10A^-^S100A9^lo^)pDC were associated with the viral persistence. The GO and KEGG analyses identified enriched pathways related to immune responses, inflammation, and apoptosis. These findings may enhance our understanding of the immunopathogenesis of COVID-19 and help develop novel strategies against SARS-CoV-2 infection.

## Introduction

The pandemic of COVID-19 has posed unprecedented challenges to the international communities. As of January 28, 2022, there have been 364,191,494 confirmed cases of COVID-19, including 5,631,457 deaths worldwide reported to the World Health Organization (1). Despite the ongoing vaccination programs, the emerging variants of SARS-CoV-2 have resulted in the devastating surge of COVID-19 cases in several regions and countries, which reminds us that we still have a tremendous task to fight the SARS-CoV-2 infections and control this devastating pandemic.

The clinical presentations of SARS-CoV-2 infection are highly variable, ranging from asymptomatic infections to critical conditions (2–13). One of these reports by a living systematic review of 86 studies in different populations and settings suggested that approximately 20%-31% of SARS-COV-2 infected individuals remained asymptomatic state (AS) during the follow-up period (5). Among the symptomatic patients (SM), approximately 80% of them showed mild or moderate diseases (MD) whereas 20% displayed severe conditions (SD). While the previous studies have suggested that both the host factors and viral mutations may contribute to the diverse manifestations of the COVID-19 (14–17), the underlying molecular mechanisms remain to be further dissected and elucidated. An increasing number of studies have demonstrated the involvement of T cells, B cells, NK cells, monocytes, neutrophils, and inflammatory macrophages in the pathogenesis of COVID-19 patients with moderate or severe diseases (18–25), suggesting that host immune responses play important roles in the pathogenesis of COVID-19. On the other hand, only a few studies have reported the immune responses in the asymptomatic individuals (AS) (26–29). It is conceivable that examining the differences in the immune responses between the AS subjects and the SM patients may help understand the protective and pathogenic immune responses to SARS-CoV-2 infection. In addition, some of the hospitalized patients were tested positive for the nucleic acid of SARS-CoV-2 by RT-PCR but did not become negative for a longer period of time (>45 days, herein designated as long-term nucleic acid test positive, LTNP) whereas others turned into negative for the viral nucleic acid in a shorter period of time (≤45 days, designated as short-term nucleic acid test positive, STNP). However, the immune cells alternations in the LTNP and STNP patients remain largely unknown.

In this study, we employed the scRNA-seq technology coupled with a deep and comprehensive analysis of unique cell surface markers and differentially expressed genes to profile 51 PBMC samples from eleven HC individuals, five AS subjects and 33 SM patients. We identified 76 distinct immune cell subsets in the PBMC samples and revealed a large number of distinct immune cell subsets that were associated with various clinical presentations and viral persistence in the COVID-19 patients. These findings have shed new light on understanding the immunopathogenesis of COVID-19 and may help develop novel strategies against SARS-CoV-2 infection.

## Materials and Methods

### Patient Cohort and Case Definition

**Figure 1:**
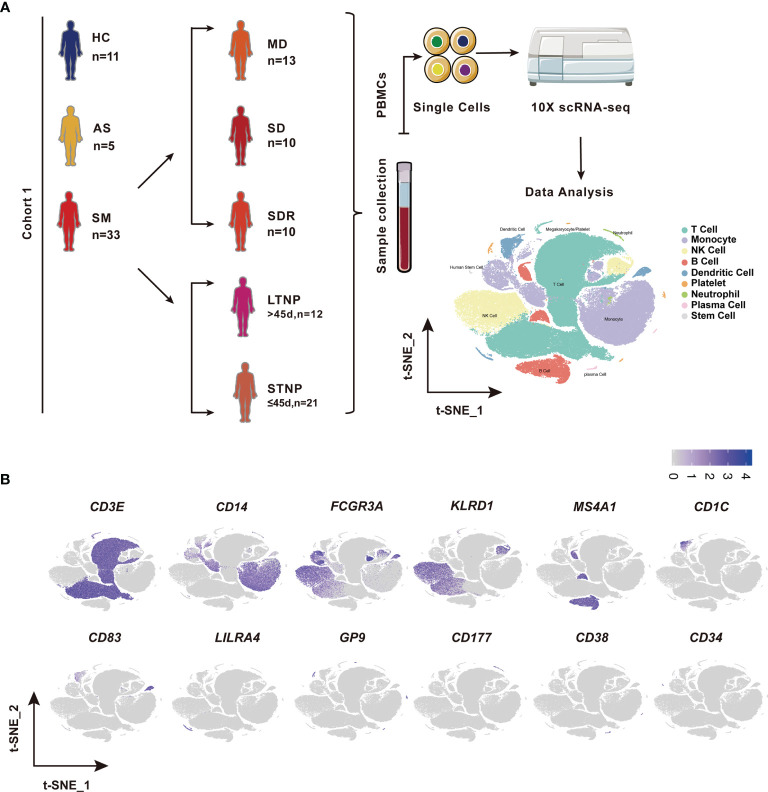
Identification of nine cell types in the peripheral blood mononuclear cells (PBMCs). **(A)** A schematic diagram of the study design for single-cell RNA sequencing (scRNA-seq). Peripheral blood mononuclear cells (PBMCs) were collected from eleven healthy controls (HC) and 39 SARS-CoV-2 infected individuals followed by scRNA-seq using the 10x Genomics platform. The T-distributed Stochastic Neighbor Embedding (t-SNE) plot of 119,799 cells showing nine cell types (i.e., T cells, Monocytes, NK cells, B cells, Dendritic cells, platelets neutrophils, plasma cells, and stem cells) identified from this study. **(B)** The t-SNE plots showing the expression of canonical cell marker genes used for identification of the nine cell types described above. Each dot represents a single cell. The plots are color-labelled based on the expression level of the respective marker gene in log scale, which was calculated *via* LogNormalize method of the “NormalizeData” function of the Seurat software. HC, Healthy controls; AS, Asymptomatic subjects; SM, Symptomatic patients; MD, Moderate disease; SD, Severe disease; SDR, Recovery patients from SD; LTNP, Long term nucleic acid test positive; STNP, Short term nucleic acid test positive.

This study was reviewed and approved by the Ethics Committee of The First affiliated Hospital of Xi’an Jiaotong University (XJTU1AF2020LSK-015) and The Renmin Hospital of Wuhan University (WDRY2020-K130). All participants enrolled in this study offered the written informed consent by themselves or their surrogates. The definition and classification of all COVID-19 patients in this study follow the Guidelines of the World Health Organization and the “Guidelines on the Diagnosis and Treatment of the Novel Coronavirus Infected Pneumonia” developed by the National Health Commission of People’s Republic of China (30–32). We collected 53 samples of peripheral blood mononuclear cells (PBMCs), including 42 COVID-19 patient samples and 11 healthy controls (HC) samples (**Figure 1A** and **Table S1**). Two samples were excluded in this study, of which COV077 was a fatal case whereas COV166 was an immunocompromised case. The number of patients included five asymptomatic subjects (AS, n=5) and 33 symptomatic patients (SM, n=33) consisting of 13 moderate disease (MD, n=13), 10 severe disease (SD, n=10), and 10 SD recovery (SDR, n=10), and two samples collected at two different time points during hospitalization from patient C-19 and C-26, respectively. The SM group was further divided into the long-term nucleic acid test positive (LTNP, n=12) and the short-term nucleic acid test positive (STNP, n=21) sub-groups. In this study, based on the clinical observation that most of the COVID-19 patients hospitalized in the Renmin Hospital in Wuhan became negative for the nucleic acid test within 45 days, we therefore defined the STNP was ≤45 days whereas the LTNP was >45 days (**Table S1**). The demographic features, clinical laboratory testing results and other relevant information were provided in **Table S1**.

### Preparation of Single-Cell Suspensions

The frozen PBMCs were retrieved from the liquid nitrogen storage tank and thawed in a 37°C water bath, followed by washing with 10mL of 90% DMEM+10% FBS in a 15-mL polypropylene tube and then centrifuged at 500g for 20 min. The supernatant was removed (repeated this step twice). The cell pellet was resuspended with 500μl of 1x PBS with 0.04% BSA (A1933-25G, SIGMA), followed by adding 5ml of 1x Red blood cell (RBC) lysis buffer (130-094-183, 10x stock solution, Miltenyi Biotech) and incubated at room temperature for 10 min to lyse the remaining red blood cells. After incubation, the cell suspension was centrifuged at 500g for 20 min at room temperature and then resuspended in 100μl of Dead Cell Removal MicroBeads to remove dead cells using Miltenyi Dead Cell Removal Kit (130-090-101-1, Miltenyi Biotech). The live cells were collected and resuspended in 1x PBS with 0.04% BSA and centrifuged at 300g for 3 min at 4°C (repeated twice). The cell pellet was resuspended in 50μl of 1x PBS with 0.04% BSA. The cell viability was measured by the trypan blue exclusion method and confirmed to be 85% or higher. The cell number of the single cell suspension was counted using a Countess II Automated Cell Counter and the final concentration was adjusted to 700-1200 cells/μl.

### Chromium 10x Genomics Library Construction and Sequencing

Approximately 5000 single cells each sample were captured using the Chromium Single-Cell 5’ kit (V1) according to the manufacturer’s instructions (PN-1000020, 10x Genomics), followed by cDNA amplification and library construction performed according to the standard protocols. The libraries were sequenced on an Illumina NovaSeq 6000 sequencing platform (paired-end multiplexing run, 150bp) by LC-Bio Technology Co. Ltd., (HangZhou, China) at a minimum depth of 20,000 reads per cell. To avoid batch effects, the scRNA-seq data sets were generated by the same operators at the same laboratories using the standard operation protocols (SOPs) for cell dissociation, library preparation and sequencing.

### Single-Cell RNA-Seq Data Processing

The sequencing results were demultiplexed and converted to FASTQ format using Illumina Bcl2fastq2 software (v2.20, Illumina). Sample demultiplexing, barcode processing and single-cell 5’ gene counting were completed by using the Cell Ranger pipeline (https://support.10xgenomics.com/single-cellgeneexpression/software/pipelines/latest/what-is-cell-ranger, version 3.1.0) and the scRNA-seq data were aligned to Ensembl genome GRCh38 reference genome. A total of 222,457 single cells captured from eleven healthy controls and 42 COVID-19 patient samples were processed using 10x Genomics Chromium Single Cell 5’ Solution (PN-1000006, 10x Genomics). The Seurat (version 3.1.1) was used for dimensional reduction, clustering, and analysis of scRNA-seq data (33). Overall, 207,718 cells passed the quality control threshold: all genes expressed in less than one cell were removed, number of genes expressed per cell >500 as low cut-off, UMI counts less than 500, and the percentage of mitochondrial-DNA derived gene-expression < 25%. To visualize the data, we further reduced the dimensionality of all 207,718 cells by Seurat and used t-Distributed Stochastic Neighbor Embedding (t-SNE) to project the cells into 2D space. The steps included: (1) Using the LogNormalize method of the “Normalization” function of the Seurat software to calculate the expression level of genes; (2) The principal component analysis (PCA) was performed using the normalized expression level, within all the PCs, the top 10 PCs were used to do clustering and t-SNE analysis; (3) Using weighted Shared Nearest Neighbor (SNN) graph-based clustering method to find clusters. The marker genes for each cluster were identified with the “bimod”(Likelihood-ratio test) with default parameters *via* the FindAllMarkers function in Seurat. This selects marker genes that were expressed in more than 10% of the cells in a cluster and the average log (Fold Change) of greater than 0.26. To further avoid interference of putative multiplets (where more than one cell was loaded into a given well on an array), cells in a defined cluster that had high expression of more than one cell type canonical marker gene were filtered to ensure the data quality. In detail, we identified nine major cell types using the canonical markers (T cell: CD3E^+^; NK: KLRD1(CD94)^+^ or CD16A^+^; Monocyte: CD14^+^ or CD16A^+^ or LYZ^+^; B cell: CD20 (MS4A1)^+^; Dendritic cells: CD16A^+^ or CD83^+^ or LILRA4(CDF85g) or LYZ^+^ or CD1C^+^; Platelet: GP9^+^ or ITGA2B (CD41)^+^; Neutrophil: CD177^+^ or LYZ^+^; Plasma cell:CD38^+^; Stem cell:CD34^+^) and then excluded any cells that expressed more than one canonical marker genes, which could not be classified into one type. Parameters used for graph-based clustering follow: FindNeighbors with parameter reduction = pca, dims = 1:10 and FindClusters with parameter resolution = 0.8. “Cellranger aggr” in Seurat was used to integrate the samples. As a result, a total of 119,799 cells were used for the final analysis in this study. The nine cell types were integrated for further sub-clustering. After integration, genes were scaled to unit variance. Scaling, principal component analysis and clustering were performed as described above.

### Analysis of Differentially Expressed Genes and Functional Enrichment

The analysis of differentially expressed genes (DEGs) between each pair of cells from different groups (e.g., the asymptomatic, symptomatic, and healthy control groups) was performed using “bimod” with default parameters in Seurat. DEGs were filtered using a minimum log2 (fold change) of 0.26, a P value <0.05 and >10% of cells expressed in at least one group. To further understand the associations and function of the DEGs, GO and KEGG pathway analysis was performed using the OmicStudio tools at https://www.omicstudio.cn/tool. DEGs with a log2 mean expression difference ≥0.26 enriched in GO or KEGG pathways were considered as significant candidate biomarkers or pathways.

### Clinical and Laboratory Tests

All clinical and laboratory tests were conducted in the Renmin Hospital of Wuhan University, including the tests of SARS-COV-2-specific IgM (Cat#20203400769, YHLO Biotech) and SARS-COV-2-specific IgG (Cat#20203400770, YHLO Biotech) antibodies.

### Statistical Analysis

All data and statistical analyses were performed using SPSS (Statistical Package for the Social Sciences, Version 23.0 software, SPSS Inc.). R (https://www.cran.r-project.org, Vienna, Austria) and GraphPad Prism 8.0.2 (GraphPad Software, San Diego, USA) were also used for analysis in this study. Categorical variables were described as frequency rates and percentages, whereas continuous variables were described using mean, median, and inter quartile range (IQR) values. Difference analysis of HC vs AS and STNP vs LTNP were conducted using two-groups comparison strategy, whereas multiple groups comparison strategy was employed for analysis of HC vs AS vs SM and HC vs AS vs MD vs SD vs SDR. The cell cluster analysis was performed using cell abundance data to identify the distinct cell subsets associated with various clinical presentations. The gene expression analysis was performed to identify the differential expression genes (DEGs). We also analyzed the data distribution in each group. Independent group t-tests were performed for two-groups comparisons when the data were normally distributed; otherwise, the Mann-Whitney test was used. For three-groups comparison, One-way Anova test was conducted when the data were normally distributed and homoscedasticity, otherwise, Kruskal-Wallis was employed. In addition, Bonferrion correction was used for the multiple- groups comparison. Of note, Bonferrion correction (Bonferroni adjustment) include the following steps: First, divide the desired alpha-level by the number of comparisons; Second, calculate the p-value and evaluate the significance. SPSS employed a mathematically equivalent adjustment in this study for pairwise comparisons. The Bonferrion correction was performed by taking the observed (uncorrected) p-value and multiply it by the number of comparisons. Proportions for categorical variables were compared using the χ2 test, whereas the Fisher exact test was employed when the data were limited. For unadjusted comparisons, a two-sided α of less than 0.05 was considered statistically significant. Correlation analyses were performed using Spearman, whereas Mann-Whitney and Wilcoxon tests were employed for unpaired and paired comparisons, respectively. The details of the statistical analysis were provided in the respective figure legends.

## Results

### Identification of 76 Cell Subsets in the PBMCs of COVID-19 Patients by scRNA-Seq

**Figure 2:**
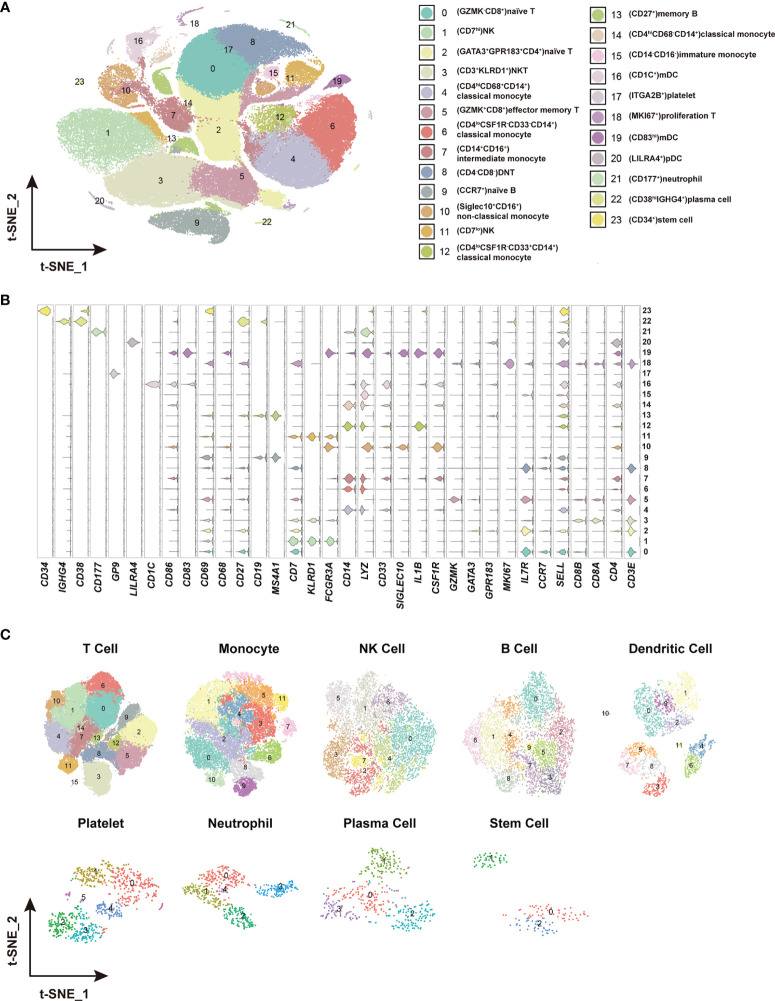
Identification of distinct cell clusters and sub-clusters in the PBMCs. **(A)** The t-SNE plot showing 24 cell clusters with separate color labels and marked by signature gene. **(B)** The violin plots showing the expression pattern of the selected cell markers in each of the 24 clusters. The marker gene expression level was calculated *via* LogNormalize method of the “NormalizeData” function of the Seurat software. **(C)** The t-SNE plots showing the sub-clusters of T cells, monocytes, NK cells, B cells dendritic cells, platelets, neutrophils, plasma cells and stem cells.

To identify the immune cell alternations in the peripheral blood of COVID-19 patients with various clinical presentations, we performed the droplet-based scRNA-seq to profile the immune cell landscape in 51 PBMC samples collected from eleven healthy controls (HC), five asymptomatic individuals (AS), and 33 symptomatic patients (SM) with moderate diseases (MD, n=13) or severe diseases (SD, n=10), and the patients recovered from SD (SDR, n=10), as well as three samples collected at two different time points during hospitalization from patient C-19 and C-26, respectively (**Figure 1A**). In addition, this study cohort included 12 long-term nucleic acid test positive (LTNP) patients, 21 short-term nucleic acid test positive (STNP) patients. (**Figure 1A** and **Table S1**). The demographic features, clinical characteristics and the laboratory testing results of these study subjects were presented in the **Supplementary Materials** (**Table S1**). After a series of stringent high-quality filtering and removal of multiplets, a total of 119,799 single cells captured from all the participants were used for the final data analysis. As shown by the t-Distributed Stochastic Neighbor Embedding (t-SNE) plot, we first identified nine major cell types, including T cells (*CD3E*), B cells (*MS4A1*), monocytes (*CD14, FCGR3A*), natural killer (*KLRD1*), dendritic cells (*CD1C, CD83, LILRA4*), platelets (*GP9*), neutrophils (*CD177*), plasma cells (*CD38*), and stem cells (*CD34*) using the unique marker genes (**Figures 1A, B**). We also examined the distribution patterns of these cell types in each of the study sub-groups (**Figure S1A**) and calculated the relative abundance of these cell types in each sample, respectively (**Figure S1B**). We then identified 24 cell clusters from the 119,799 single cells, including (CD8^+^GZMK^-^)naïve T cells (cluster 0), (CD7^hi^)NK cells (cluster 1), (CD4^+^GATA3^+^GPR183^+^) naïve CD4 T cells (cluster 2), (CD3^+^KLRD1^+^)NKT cells (cluster 3), (CD4^hi^CD68^+^CD14^+^) classical monocytes (cluster 4), (GZMK^+^CD8^+^)effector/memory CD8 T cells (cluster 5), (CD4^lo^CSF1R^-^CD33^-^CD14^+^)classical monocytes (cluster 6), (CD14^+^CD16^+^)intermediate monocytes (cluster 7), (CD4^-^CD8^-^)double negative T cells (cluster 8), (CCR7^+^)naïve B cells (cluster 9), (Siglec10^+^CD16^+^)non-classical monocytes (cluster 10), (CD7^lo^)NK cells (cluster 11), (CD4^lo^CSF1R^-^CD33^+^CD14^+^)classical monocytes (cluster 12), (CD27^+^)memory B cells (cluster 13), (CD4^hi^CD68^-^CD14^+^)classical monocytes (cluster 14), (CD14^-^CD16^-^)immature monocytes (cluster 15), (CD1C^+^)myeloid DC (mDC) (cluster 16), (ITGA2B^+^)platelets (cluster 17), (MKI67^+^)proliferation T cells (cluster 18), (CD83^hi^)mDC (cluster 19), (LILRA4^+^)plasmacytoid DC (pDC) (cluster 20), (CD177^+^)neutrophils (cluster 21), (CD38^hi^IGHG4^+^)plasma cells (cluster 22), and (CD34^+^)stem cells (cluster 23) (**Figures 2A–C** and **Table S2**), and calculated the relative abundance of the 24 cell clusters in each of the samples, respectively (**Figure S2A**). Furthermore, we identified 76 distinct cell subsets from the nine cell types with a combination of unique cell surface markers and the differentially expressed genes, including sixteen T cell subsets, twelve monocyte subsets, twelve dendritic cell subsets, eight NK cell subsets, ten B cell subsets, six platelet subsets, five neutrophil subsets, four plasma cell subsets, and three stem cell subsets (**Figures 2B, C**, **S2B**, and **Table S2**). We also calculated the relative abundance of the cell types, clusters and subclusters described above (**Tables S3** and **S4**), which allowed us to compare the cell alternations between various groups of individuals and understand their associations with the pathogenesis of COVID-19.

### Distinct Immune Cell Subsets Associated With Asymptomatic Infection of SARS-COV-2

**Figure 3:**
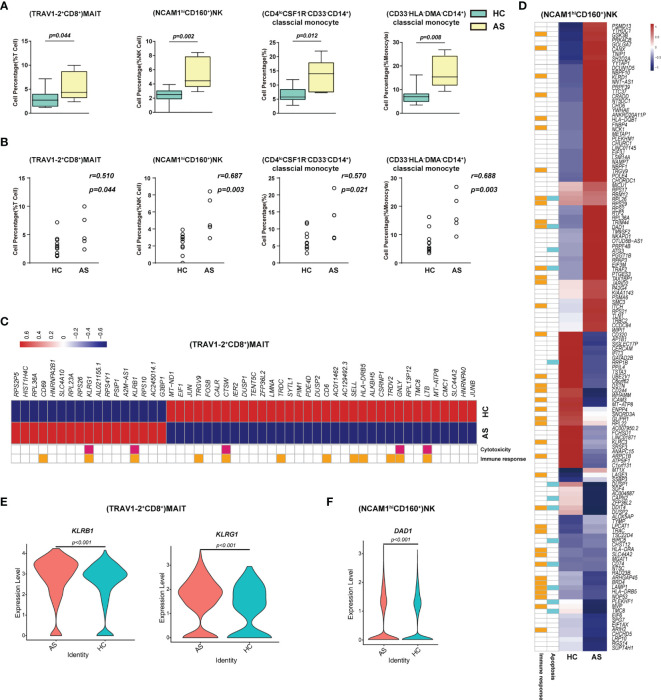
Immune cell subsets associated with asymptomatic COVID-19 patients. **(A)** The Box and whisker plots showing the percentage of four cell clusters that had significant differences (p < 0.05) HC and AS groups. The horizontal lines, box and whiskers correspond to median values, interquartile range (IQR) and minimum/maximum value, respectively. **(B)** Spearman rank order correlation analysis showing the percentage of the four cell types described above are positive associated with the asymptomatic state **(C)** A Heatmap showing the average level of the differentially expressed genes (DEGs) in the (TRAV1-2^+^CD8^+^)MAIT cells with p < 0.05 and log2 fold change (FC) ≥0.26 in HC and AS. The gene function (immune responses and cytotoxicity) is indicated in the heatmap. The gene expression was calculated *via* LogNormalize method of the “NormalizeData” function of the Seurat software. **(D)** A Heatmap showing the average level of the DEGs in the (NCAM1^hi^CD160^+^)NK cells with p < 0.05 and log2 fold change (FC) ≥0.26 in HC and AS. The gene function (immune responses and apoptosis) is indicated in the heatmap. The gene expression was calculated *via* LogNormalize method of the “NormalizeData” function of the Seurat software. **(E)** The violin plot showing the expression levels of two representative DEGs (*KLRB1* and *KLRG1*) in (TRAV1-2^+^CD8^+^)MAIT cells, which was involved in cytotoxicity and/or immune responses. **(F)** The violin plot showing the expression levels of one representative DEG (*DAD1*) in (NCAM1^hi^CD160^+^)NK cells, which was involved in apoptosis and/or immune responses (also see **Figure S3**). p < 0.05 was considered significant. The samples included HC (n=11) and AS (n=5).

To understand the protective and pathogenic immune responses to SARS-CoV-2 infection, we first compared the percentage of each cell type between the healthy controls (HC) and the asymptomatic subjects (AS). We found that (TRAV1-2^+^CD8^+^)MAIT cells, (NCAM1^hi^CD160^+^) NK cells, (CD4^lo^CSF1R^-^CD33^-^CD14^+^)classical monocytes and (CD33^-^HLA-DMA^-^CD14^+^) classical monocytes increased significantly in the AS group and were positively correlated with the AS (**Figures 3A, B** and **Table 1**). We further analyzed the differentially expressed genes (DEGs) in these cell subsets (**Table S5**). The heatmap in **Figure 3C** showed the average level of 51 DEGs in the (TRAV1-2+CD8+)MAIT cells from the HC and AS individuals, of which twelve genes (such as *CD6*, *CD69* and *KLRB1*) were related to immune responses, and five genes (such as *KLRB1*, *KLRG1*, and *GNLY*) were linked to cytotoxicity. Of note, the Killer Cell Lectin Like Receptor B1(KLRB1) and the Killer Cell Lectin Like Receptor G1 (*KLRG1*), which have been suggested to be involved in innate immune responses, NK cell-mediated cytotoxicity and T cell activations (34, 35), were overexpressed in the AS subjects (**Figure 3E**). The heatmap in **Figure 3D** showed the average level of 134 DEGs in the (NCAM1^hi^CD160^+^)NK cells from the HC and AS individuals, of which 39 genes (such as *GSK3B*, *CANX* and *KLRD1*) were related to immune responses, and 13 genes (such as *DAD1*, *ATG3*, and *TRAF2*) were linked to apoptosis pathways (**Figure 3C**). Of note, the Defender Against Cell Death 1 (*DAD1*) was overexpressed in the AS subjects (**Figure 3F**). Additional analysis of Gene Ontology (GO) and KEGG pathways revealed significant enrichments in viral transcription (**Figure S3C**). These findings suggest that the innate immune responses may play important roles in controlling the SARS-CoV-2 infections in the AS individuals.

**Table 1:** Distinct cell subsets associated with various clinical presentations of SARS-CoV-2 infection.

Cell types and subsets	AS vs HC	SM vs AS	SD vs MD	LTNP vs STNP
(CD4^-^CD8^-^)DNT		↓	↓	
(TRGC1^-^CD4^-^CD8^-^)DNT			↓	
(AIF1^+^BCL2^+^CD8^+^)naïve T				↓
(GATA3^+^CCR6^-^S1PR1^+^CD4^+^)naïve T		↑	↑	
(GATA3^+^GPR183^+^CD4^+^)naïve T		↑	↑	
(MKI67^+^)Proliferation T			↑	↓
(TRGC1^+^CD4^-^CD8^-^)γδT			↓	
(TRAV1-2^+^CD8^+^)MAIT	↑		↓	
(FOXP3^+^IL2RA^+^IL7R^+^CD4^+^)Treg		↑	↑	
(LAG3^+^CD160^+^CD8^+^)NKT		↑	↑	
(LAMP1^+^CD4^+^CD8^+^)pro-NKT			↑	
(CD7^lo^)NK		↓	↓	
(NCAM1^hi^CD160^+^)NK	↑		↑	
B cells			↓	
(CD24^-^ITGAE^+^CD180^+^) marginal zone B cells				↓
(IGHD^+^CD27^+^CD180^-^) memory B		↓		
Plasma cells				↓
(CD68^-^CSF1R^-^IL1B^hi^CD14^+^) classical monocytes			↑	↓
(CD4^lo^CSF1R^-^CD33^-^CD14^+^) classical monocytes	↑	↓		
(CD4^lo^CSF1R^-^CD33^+^CD14^+^) classical monocytes			↑	↓
(CD33^-^HLA-DMA^-^CD14^+^) classical monocytes	↑	↓	↓	↑
(CSF1R^+^CD86^-^CD14^+^) classical monocytes		↓	↓	
(CXCL8^+^CSF1R^-^IL1B^-^CD14^+^) classical monocytes		↓	↓	↑
(CD14^lo^CD16^lo^)immature monocytes			↓	↑
(CD40^+^CLEC9A^-^)mDC			↑	
(CD83^hi^)mDC			↑	
(CX3CR1^hi^CD14^hi^)mDC			↑	
(S100A12^-^CX3CR1^lo^)mDC			↓	
(TNFRSF8^+^)mDC			↑	
(CLEC10A^-^S100A9^lo^)pDC			↓	↑
Neutrophils				↑
(MMRN1^-^PDZK1IP1-) activated platelets				↓

HC, Healthy controls; AS, Asymptomatic subjects; SM, Symptomatic patients; LTNP, long-term nucleic acid test positive patients; STNP, short-term nucleic acid test positive patients; hi, high; lo, low; ↑= cell percentage increased and/or showing positive correlation; ↓=cell percentage decreased and/or showing negative correlation.

### Immune Cell Subsets Associated With the Symptomatic COVID-19 Patients

**Figure 4:**
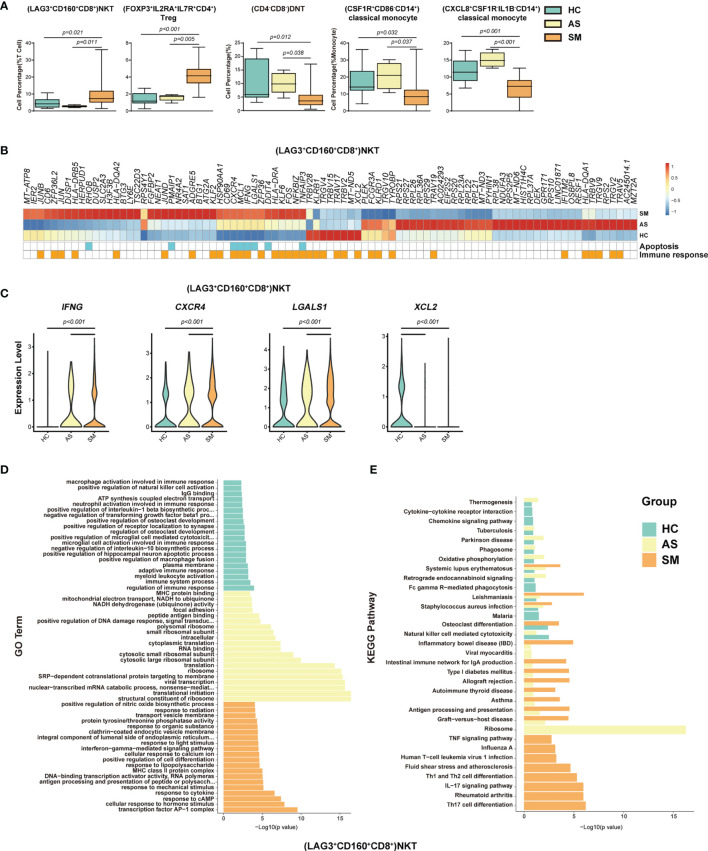
Immune cell subsets associated with symptomatic COVID-19 patients. **(A)** The Box and whisker plots showing the percentage of five distinct cell clusters that exhibited significant differences (p < 0.05) between HC, AS, and SM groups. The horizontal lines, box and whiskers correspond to median values, interquartile range (IQR) and minimum/maximum value, respectively. **(B)** A Heatmap showing the average level of the DEGs in the (LAG3^+^CD160^+^CD8^+^)NKT cells with p < 0.05 and log2 fold change (FC) ≥0.26 in HC, AS and SM. The gene function (immune responses and apoptosis) is indicated in the heatmap. The gene expression was calculated *via* LogNormalize method of the “NormalizeData” function of the Seurat software. **(C)** The violin plots showing the expression levels of four representative DEGs (*IFNG, CXCR4, LGALS1*, and *XCL2*) in (LAG3^+^CD160^+^CD8^+^)NKT cells, which were involved in apoptosis and/or immune responses. **(D)** Gene Ontology (GO) analysis. Top 20 significant GO terms sorted by − Gene Ontology (GO) analysis. Top 20 significant GO ter^+^CD160^+^CD8^+^)NKT cells. **(E)** KEGG pathway analysis. Top 20 significant KEGG pathways sorted by −log10 (p value) from HC, AS, and SM were shown in (LAG3^+^CD160^+^CD8^+^)NKT cells (also see **Figure S3**). p < 0.05 was considered significant. The samples included HC (n=11), AS (n=5) and SM (n=35, total 33 patients and two patients were sampled twice at different disease stages).

To identify the potential cell subsets that may contribute to the development of clinical symptoms in COVID-19 patients, we compared the percentage of each cell type between the HC, AS and symptomatic (SM) groups, respectively, and detected 21 cell subsets that either increased or decreased significantly in these groups (**Figures 4A**, **S3A** and **Table 1**). In particular, we observed that (LAG3+CD160+CD8+)NKT cells, (FOXP3^+^IL2RA^+^ IL7R^+^CD4^+^)Treg cells, (GATA3^+^GPR183^+^CD4^+^)naïve T cells, (GATA3^+^CCR6^-^S1PR1^+^CD4^+^)naïve T cells, (MKI67^+^)proliferation T cells, (CCR6^+^CD4^+^)Th17 cells, and (LAMP1^+^ CD4^+^CD8^+^)pro-NKT cells increased significantly whereas (CD4^-^CD8^-^)DNT (the double negative T cells), (CSF1R^+^CD86^-^CD14^+^)classical monocytes, (CXCL8^+^CSF1R^-^IL1B^-^CD14^+^)classical monocytes, (AIF1^+^BCL2^+^CD8^+^)naïve T cells, (TRGC1^+^CD4^-^CD8^-^)γδT cells, (CD4^lo^CSF1R^-^CD33^-^CD14^+^)classical monocytes, (CD68^+^IL1B^lo^CD14^+^)classical monocytes, (CD33^-^HLADMA^-^CD14^+^)classical monocytes, (CD7^lo^)NK cells, (IGHD^+^CD27^+^ CD80^-^)memory B cells, (CD1C^+^)mDC, (LILRA4^+^)pDC, and (CLEC10A^-^S100A9^-^)pDC decreased significantly in the SM patients compared with the AS subjects (**Figures 4A** and **S3A**). We further analyzed the differentially expressed genes (DEGs) in these cell subsets to understand the immune responses and pathogenesis of SARS-CoV-2 infection. Here, we showed a representative heatmap with the average level of 89 DEGs in the (LAG3+CD160+CD8+)NKT cells from the HC, AS and SM individuals, of which 35 genes (e.g. *CXCR4*, *IFNG* and *XCL2*) were related to immune responses, and eight genes (*RHOB, PMAIP1*, *CXCR4, MCL1, IFNG, LGALS1, DDIT4* and *TNFAIP3*) were linked to apoptosis pathways (**Figures 4B, C**). Additional analysis of Gene Ontology (GO) and KEGG pathways revealed a number of DEGs that were associated with viral transcription, response to cytokines, interferon-gamma-mediated signaling pathway, TNF signaling pathway, Th1 and Th2 cell differentiation, Th17 cell differentiation, antigen processing and presentation, and other pathways (**Figures 4E, F** and **S3B–D**). Of note, all the genes associated with the apoptosis pathways were up-regulated in the SM group, indicating that (LAG3+CD160+CD8+)NKT cells may play a critical role in the pathogenesis of COVID-19.

### Immune Cell Subsets Associated With the Disease Severity of COVID-19

**Figure 5:**
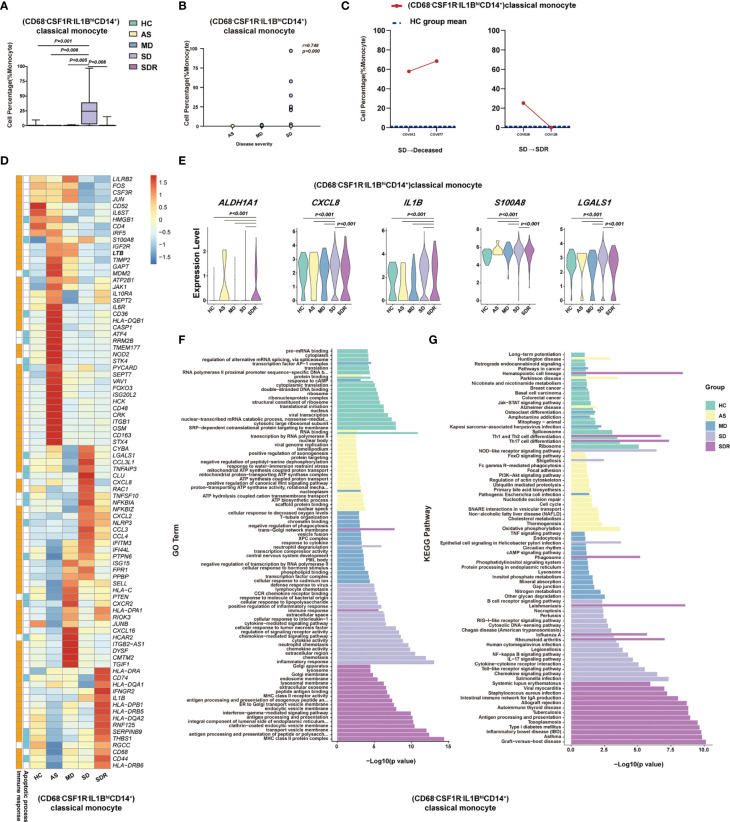
(CD68-CSF1R-IL1BhiCD14+)classical monocyte correlated with disease severity of COVID-19 patients. **(A)** The Box and whisker plots showing the percentage of (CD68^-^CSF1R^-^IL1B^hi^CD14^+^)classical monocytes that showed significant differences (p< 0.05) between the HC, AS, MD, SD and/or SDR groups. The horizontal lines, box and whiskers correspond to median value, interquartile range (IQR) and minimum/maximum value, respectively. **(B)** Spearman rank order correlation analysis showing the significant associations between (CD68^-^CSF1R^-^IL1B^hi^CD14^+^)classical monocytes with the disease severity. **(C)** The alternation trend of (CD68^-^CSF1R^-^IL1B^hi^CD14^+^)classical monocytes in the fatal patient (COV012/COV077) and one recovery SD patient (COV029/COV126). **(D)** The Heatmap showing the average level of the DEGs in the (CD68^-^CSF1R^-^IL1B^hi^CD14^+^)classical monocytes with p< 0.05 and log2 fold change (FC) ≥0.26 in HC, AS, MD, SD, and SDR. The gene function (immune responses and apoptosis) is indicated in the heatmap. The gene expression was calculated *via* LogNormalize method of the “NormalizeData” function of the Seurat software. **(E)** The violin plots showing the expression levels of five representative DEGs (*ALADH1A1, CXCL8, IL1B, S100A8*, and *LGALS1*) in (CD68^-^CSF1R^-^IL1B^hi^CD14^+^)classical monocytes, which were involved in apoptosis and/or immune responses. **(F)** Gene Ontology (GO) analysis. Top 20 significant GO terms sorted by −log10 (p) value from HC, AS, MD, SD, and SDR were shown, respectively. **(G)** KEGG pathway analysis. Top 20 significant KEGG pathways sorted by −log10 (p value) from HC, AS, MD, SD, and SDR were shown, respectively (also see **Figure S4**). p< 0.05 was considered significant. The samples included HC (n=11), AS (n=5), MD (n=13), SD (n=11, one patient was sampled twice at different disease stages) and SDR (n=11, one sample was sampled at different disease stages).

To delineate the immune cell subsets associated with the disease severity of COVID-19, we compared the percentage of each cell type, cluster and sub-cluster between HC, AS, MD, SD, and SDR, respectively. We identified 27 distinct cell subsets that increased or decreased significantly in these groups, 23 of which were positively or negatively correlated with the disease severity by the Spearman rank order correlation analysis (**Figures 5A, B**, **S4A, B** and **Table 1**). When we compared the SD with MD, we observed that (GATA3^+^CCR6^-^S1PR1^+^CD4^+^)naïve T, (GATA3^+^GPR183^+^CD4^+^)naïve T, (MKI67^+^)Proliferation T, (FOXP3^+^IL2RA^+^IL7R^+^CD4^+^)Treg, (LAG3^+^CD160^+^CD8^+^)NKT, (LAMP1^+^CD4^+^CD8^+^)pro-NKT, (NCAM1^hi^CD160^+^)NK, (CD68^-^CSF1R^-^IL1B^hi^CD14^+^)classical monocytes, (CD4^lo^CSF1R^-^CD33^+^CD14^+^)classical monocytes, (CD40^+^CLEC9A^-^)mDC, (CD83^hi^)mDC, (CX3CR1^hi^CD14^hi^)mDC and (TNFRSF8^+^)mDC increased significantly, whereas (CD4^-^CD8^-^)DNT, (TRGC1^-^CD4^-^CD8^-^)DNT, (TRGC1^+^CD4^-^CD8^-^)γδT, (TRAV1-2^+^CD8^+^)MAIT, (CD7^lo^)NK, (CD33^-^HLA-DMA^-^CD14^+^)classical monocytes, (CSF1R^+^CD86^-^CD14^+^)classical monocytes, (CXCL8^+^CSF1R^-^IL1B^-^CD14^+^)classical monocytes, (CD14^lo^CD16^lo^)immature monocytes, (S100A12^-^CX3CR1^lo^)mDC, and (CLEC10A^-^S100A9^lo^)pDC decreased significantly (**Figures 5A, B**, **S4A, B**, and **Table 1**). Here, we showed (CD68^-^CSF1R^-^IL1B^hi^CD14^+^)classical monocytes as a representative cell subset that was positively correlated with the disease severity (**Figures 5A, B**).

To investigate whether (CD68^-^CSF1R^-^IL1B^hi^CD14^+^)classical monocytes were associated with the disease progression, we examined two SM patients who had PBMC samples collected at two different time-points, including patient #1 (a fatal patient): COV012 (time-point 1) and COV077 (time-point 2); and patient #2 (an SD patient at the recovery stage): COV029 (time-point 1) and COV126 (time-point 2). We observed that (CD68^-^CSF1R^-^IL1B^hi^CD14^+^)classical monocytes dramatically increased in both time points in the fatal patient (COV012→ COV077), and the second time point was higher than the first one (**Figure 5C**). In contrast, for the SD patient at the recovery stage (SDR) (COV029→COV126), the percentage of (CD68^-^CSF1R^-^IL1B^hi^CD14^+^)classical monocytes at the second time point resumed to the level similar to the healthy control (HC) group (**Figure 5C**), suggesting that (CD68^-^CSF1R^-^IL1B^hi^CD14^+^)classical monocytes may play an important role in the pathogenesis of COVID-19.

We also analyzed the DEGs in these cell subsets and presented a representative heatmap showing the average level of 88 DEGs in the (CD68^-^CSF1R^-^IL1B^hi^CD14^+^)classical monocytes in the HC, AS, MD, SD and SDR individuals. Most of the DEGs were related to immune responses and apoptosis pathways (**Figure 5D**), such as *ALADH1A1, CXCL8, IL1B, S100A8*, and *LGALS1* (**Figure 5E**). The DEGs were also detected in other cell subsets, such as (CD4^-^CD8^-^)DNT cells, (TRGC1^+^CD4^-^CD8^-^)γδT cells, (CD4^lo^CSF1R^-^CD33^+^CD14^+^)classical monocytes and (CLEC10A^-^S100A9^lo^)pDC (**Figure S4C, D**). Additional analysis of the GO and KEGG pathways revealed a number of DEGs that were involved in chemotaxis, inflammatory and immune responses, and the signaling pathways of chemokine, NF-kappa B, IL-17, Toll-like receptor and apoptosis (**Figures 5F, G** and **S4E, F**).

### Immune Cell Subsets Associated With the Viral Persistence of SARS-CoV-2 Infection

**Figure 6:**
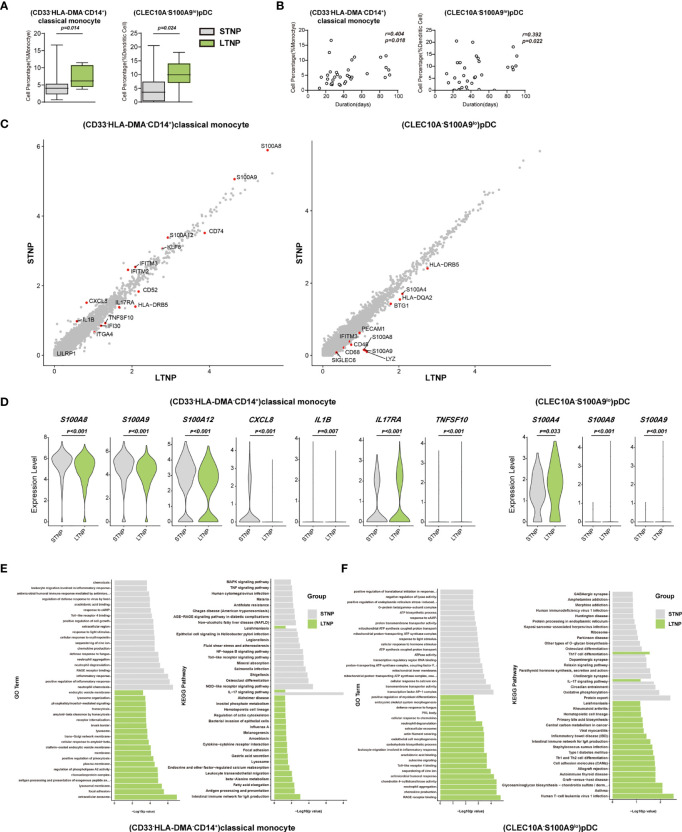
(CD33-HLA-DMA-CD14+)classical monocytes and (CLEC10A-S100A9lo)pDC were positively correlated with the viral persistence. **(A)** The Box and whisker plots showing the percentage of (CD33^-^HLA-DMA^-^CD14^+^)classical monocytes and (CLEC10A^-^S100A9^lo^)pDC that had significant differences (p < 0.05) between the STNP and LTNP groups, respectively. The horizontal lines, box and whiskers correspond to median values, interquartile range (IQR) and minimum/maximum value, respectively. **(B)** Spearman rank order correlation analysis showing the (CD33^-^HLA-DMA^-^CD14^+^)classical monocytes and (CLEC10A^-^S100A9^lo^)pDC were positively correlated with the viral persistence. **(C)** The M-versus-A (MA) plots showing the differentially expressed genes (DEGs) in the (CD33^-^HLA-DMA^-^CD14^+^)classical monocytes and (CLEC10A^-^S100A9^lo^)pDC with p < 0.05 and log2 fold change (FC) ≥0.26 in STNP and LTNP. The gene expression was calculated *via* LogNormalize method of the “NormalizeData” function of the Seurat software. The X and Y axis represent log (1+average expression value), respectively. **(D)** The violin plots showing the expression levels of seven representative DEGs (*S100A8, S100A9, S100A12, IL17RA, TNFSF10, CXCL8*, and *IL1B*) in (CD33^-^HLA-DMA^-^CD14^+^)classical monocytes and three representative DEGs (*S100A4, S100A8, S100A9*) in (CLEC10A^-^S100A9^lo^)pDC. **(E)** Gene Ontology (GO) and KEGG analysis for (CD33^-^HLA-DMA^-^CD14^+^)classical monocytes. Top 20 significant GO terms and KEGG pathways sorted by −log10 (p value) from STNP and LTNP were shown. **(F)** GO and KEGG analysis for (CLEC10A^-^S100A9^lo^) pDC. Top 20 significant GO terms and KEGG pathways sorted by −log10 (p value) from STNP and LTNP were shown (also see **Figure S5**). p < 0.05 was considered significant. The samples included LTNP (n=12) and STNP (n=21).

To understand the immune cell alternations associated with the LTNP and STNP, we compared the percentage of each of the major cell types and 76 cell subsets, and found that the LTNP subjects had significant increases in (CD33^-^HLA-DMA^-^CD14^+^)classical monocytes, (CXCL8^+^CSF1R^-^IL1B^-^CD14^+^)classical monocytes, (CD14^lo^CD16^lo^)immature monocytes, and (CLEC10A^-^S100A9^-^)pDC and neutrophils, as well as significant decreases in (AIF1^+^BCL2^-^CD4^+^)naive T cells, (MKI67^+^)proliferation T cells, (CD24^-^ITGAE^+^CD180^+^)marginal zone B cells, plasma cells, (CD68^-^CSF1R^-^IL1B^hi^CD14^+^)classical monocytes, and (CD4^lo^CSF1R^-^CD33^+^CD14^+^)classical monocytes (**Figure 6A**, **S5A**, and **Table 1**). The Spearman rank order correlation analysis suggested that (CD33^-^HLA-DMA^-^CD14^+^)classical monocytes, (CLEC10A^-^S100A9^-^)pDC, (CXCL8^+^CSF1R^-^IL1B^-^CD14^+^)classical monocytes, (CD14^lo^CD16^lo^)immature monocytes, and neutrophil were positively correlated with LTNP, whereas (CD24^-^ITGAE^+^CD180^+^)marginal zone B cells and (MMRN1^-^PDZK1IP1^-^)activated platelets were negatively correlated with LTNP (**Figure 6B**, **S5B**, and **Table 1**). Additional analysis of DEGs on two representative cell subsets found that the expression level of *S100A8, S100A9, S100A12, CXCL8, KIF6, IFITM2, IFITM3*, and *IL1B* significantly increased in STNP whereas *CD74, CD52, HLA-DRB5, IL17RA, TNFSF10, IFI30, ITGA4*, and *LILRP1* significantly increased in LTNP in the (CD33^-^HLA-DMA^-^CD14^+^)classical monocytes (**Figure 5C, D**). On the other hand, *HLA-DRB5, S100A4, HLA-DQA2, BTG1, PECAM1, IFITM3, S100A8, S100A9, CD48, CD68*, and *SIGLEC6* significantly increased in LTNP in the (CLEC10A^-^S100A9^-^)pDC (**Figures 6C, D**). The GO and KEGG analysis showed significant enrichments in antigen processing and presentation, neutrophil aggregation, chemokine production, Th1 and Th2 cell differentiation and other pathways (**Figures 6E, F**). To our knowledge, this is the first scRNA-seq study showing the immune cell alternations associated with the viral persistence of SARS-CoV-2 infection.

## Discussion

In this study, we identified a huge number of distinct cell subsets that were associated with asymptomatic infection, disease severity, and viral persistence in COVID-19 patients. In particular, we revealed that (TRAV1-2^+^CD8^+^)MAIT cells and (NCAM1^hi^CD160^+^)NK cells were significantly enriched in the AS subjects compared with the HC individuals (**Figure 4A**, **Table 1** and **Table S2**). On the other hand, (TRAV1-2^+^CD8^+^)MAIT cells were negatively correlated with the disease severity in COVID-19 patients (**Table 1**). The mucosa-associated invariant T (MAIT) cells were suggested to be associated with the mucosa immunity to a variety of microbial infections (36–39). In addition, recent studies reported that MAIT cells significantly declined in COVID-19 patients with severe diseases and resumed to normal level when the disease was resolved (40–42). These findings suggest that (TRAV1-2^+^CD8^+^)MAIT cells might be involved in the protective immune responses against SARS-CoV-2 infection.

Natural killer (NK) cells are important components of the innate and adaptive immune responses and have been suggested to play protective or pathogenic roles in the pathogenesis of human diseases, including SARS-CoV-2 infections (43–49). In this study, (NCAM1^+^CD160^+^)NK cells increased significantly in all of the SARS-CoV-2 infected individuals compared to the HC (**Figures 3A, B**, and **Table 1**) and were positively correlated with the disease severity (**Figure S4B**), suggesting that this NK cell subset may play both protective and pathogenic roles in the pathogenesis of COVID-19. In contrast, (CD7^lo^)NK cells decreased significantly in all of the SARS-CoV-2 infected individuals compared to the HC and were negatively correlated with the disease severity (**Figure S4B**), indicating that this NK cell subset may function differently from (NCAM1^+^CD160^+^)NK cells. Moreover, our results also suggest that (CD4^lo^CSF1R^-^CD33^-^CD14^+^) and (CD33^-^HLA-DMA^-^CD14^+^) classical monocytes were involved in the innate immune responses against SARS-CoV-2 infections. Therefore, it is possible that robust innate immune responses may control the virus replication and allow sufficient time to mount acquired immune responses, resulting in an asymptomatic infection and disease-free state in the AS subjects.

Natural killer T cells (NKT) and regulatory T cells (Treg) have been suggested to play important roles in immune responses to viral infections and tumors (50–58). In this study, (LAG3^+^CD160^+^CD8^+^)NKT and (FOXP3^+^IL2RA^+^IL7R^+^)Treg cells increased significantly in the SM patients (**Figure 4B**). In addition, we observed a trend towards increasing levels of these cell subsets in the SD patients. The differentially expressed genes (DEGs) in the (LAG3^+^CD160^+^CD8^+^)NKT cells (e.g. *IFNG, CXCR4, LGALS1* and *XCL2*) and (FOXP3^+^IL2RA^+^IL7R^+^) Treg cells (e.g. *CCR7, IFI16, TNFRSF4* and *TNFRSF18*), which were linked to immune responses, inflammation and apoptosis, suggest that these cells may contribute to the development of clinical symptoms and the subsequent disease progression (**Figures 4C–F** and **S3B–D**). Since (LAG3^+^CD160^+^ CD8^+^)NKT cells were also positively correlated with the disease severity, suggesting that it may function as a double edge sword, playing both protective and pathogenic roles in the pathogenesis of COVID-19.

Myeloid cell subsets, such as monocytes, macrophages, dendric cells and neutrophils have been suggested to be involved in a variety of inflammatory responses, including the pathogenesis of COVID-19 (19, 25, 59–67). In this study, we identified a large number of myeloid cell subsets that were associated with the disease severity and viral persistence in the COVID-19 patients (**Table 1**). Notably, (CD4^lo^CSF1R^-^CD33^-^CD14^+^), (CD33^-^HLA-DMA^-^CD14^+^), (CSF1R^+^CD86^-^CD14^+^) and (CXCL8^+^CSF1R^-^IL1B^-^CD14^+^) classical monocytes decreased significantly in the SM patients (**Table 1**), suggesting that these cell subsets may negatively associated with the disease symptoms. On the other hand, (CD68^-^CSF1R^-^IL1B^hi^CD14^+^) and (CD33^-^HLADMA^-^CD14^+^) classical monocytes were positively correlated with the disease severity and were associated with aggregation of neutrophils. Of note, (CD68^-^CSF1R^-^IL1B^hi^CD14^+^) and (CD33^-^HLADMA^-^CD14^+^) classical monocytes as well as neutrophils dramatically increased in the fatal patient (COV077). Moreover, these cells overexpressed a number of cytokines, chemokines, acute-phase proteins and other proinflammatory factors (e.g. *CCL3, CCL4, CXCL2, CXCL8, IL1B, S100A8, TNFSF10, LGALS1*), suggesting that these cell subsets might be associated with the cytokine storm and other pathological processes. On the other hand, (CLEC10A^-^S100A9^lo^) pDC were negatively correlated with the disease severity of COVID-19 patients.

Another novel and important aspect of this study was the profiling of PBMCs from the COVID-19 patients with short and long duration of viral persistence (i.e. STNP and LTNP). In particular, (CD33^-^HLA-DMA^-^CD14^+^)classical monocytes and (CLEC10A^-^S100A9^-^)pDC were found to be positively correlated with LTNP (**Figure 6A**). Moreover, we detected a panel of DEGs (e.g. *S100A8, S100A9, S100A12, CXCL8, KIF6, IFITM2, IFITM3*, and *IL1B*) that may discriminate the LTNP from the STNP patients (**Figures 6C, D**). HLA-DMA is a member of the HLA class II alpha chain paralogues and plays a critical role in the antigen presentations (68, 69). The enrichment of (CD33-HLA-DMA-CD14^+^)classical monocytes may weaken the antigen presentation ability and antiviral immune responses, and subsequently result in the prolonged viral persistence in the LTNP patients.

In conclusion, this study identified a large number of distinct immune cell subsets that were associated with various clinical presentations and viral persistence. Our findings may enhance our understanding of the immunopathogenesis of COVID-19. In addition, our huge datasets will become a valuable resource for future studies in the scientific communities.

## Data Availability Statement

The raw sequence data reported in this paper have been deposited in GEO repository, under accession code GSE165080 and are publicly accessible at https://www.ncbi.nlm.nih.gov/geo/query/acc.cgi?acc=GSE165080. Other supporting raw data are available from the corresponding author upon reasonable request. Source data are provided with this paper.

## Ethics Statement

This study was reviewed and approved by the Ethics Committee of The First affiliated Hospital of Xi’an Jiaotong University (XJTU1AF2020LSK-015) and The Renmin Hospital of Wuhan University (WDRY2020-K130). All participants enrolled in this study offered the written informed consent by themselves or their surrogates. The patients/participants provided their written informed consent to participate in this study.

## Author Contributions

Conceptualization: CZ and BS. Methodology and Investigation: XW, HB, JM, HQ, QZ, FH, TJ, WM, YZ, XC, XQ, ML, JX, JH, YW, XD, YL, TH, CF, CG, and CZ. Resources: CZ, BS, BHZ, and KH. Writing: CZ, BS, BJZ, XR, XW, HB, JM, TJ, and WM. Review and editing: CZ, HB, JM, BJZ, XR, and BS. Supervision: CZ, BS, BHZ, KH, CF, and DL. Funding: CZ and BS. Project administration: CZ and BS. All authors contributed to the article and approved the submitted version.

## Funding

This study is supported in part by the Department of Science and Technology of Shaanxi Province (Grant No. 2020ZDXM2-SF-02) (CZ and BS) and the operational funds from The First Affiliated Hospital of Xi’an Jiaotong University (CZ and BS).

## Conflict of Interest

The authors TJ, WM, YL, TH, CF, and CG are employed by the LC-BIO TECHNOLOGIES (HANGZHOU) CO., LTD., China.

The remaining authors declare that the research was conducted in the absence of any commercial or financial relationships that could be construed as a potential conflict of interest.

## Publisher’s Note

All claims expressed in this article are solely those of the authors and do not necessarily represent those of their affiliated organizations, or those of the publisher, the editors and the reviewers. Any product that may be evaluated in this article, or claim that may be made by its manufacturer, is not guaranteed or endorsed by the publisher.
