# Supplementary material for: Identification of Distinct Immune Cell Subsets Associated With Asymptomatic Infection, Disease Severity, and Viral Persistence in COVID-19 Patients
Source: Front Immunol. 2022 Feb 22;13:812514. doi: 10.3389/fimmu.2022.812514 (PMC8905648; doi:10.3389/fimmu.2022.812514)
Supplement: Supplementary file 1 [file DataSheet_1.pdf]

Supplementary Material

1 Supplementary Tables

Table S1. Cohort characteristic, related to Fig. 1.

Table S2. Identification results of cluster and subcluster, related to Fig. 1.

Table S3. Cell percent datasets, related to Fig. 1-7.

Table S4. Cell count datasets, related to Fig. 1.

Table S5. DEGs analysis results, related to Fig. 2-6.

Table S6. KEGG pathway analysis results, related to Fig. 2-6.

Table S7. GO Term analysis results, related to Fig. 2-6.

2 Supplementary Figures

Supplementary Figure 1

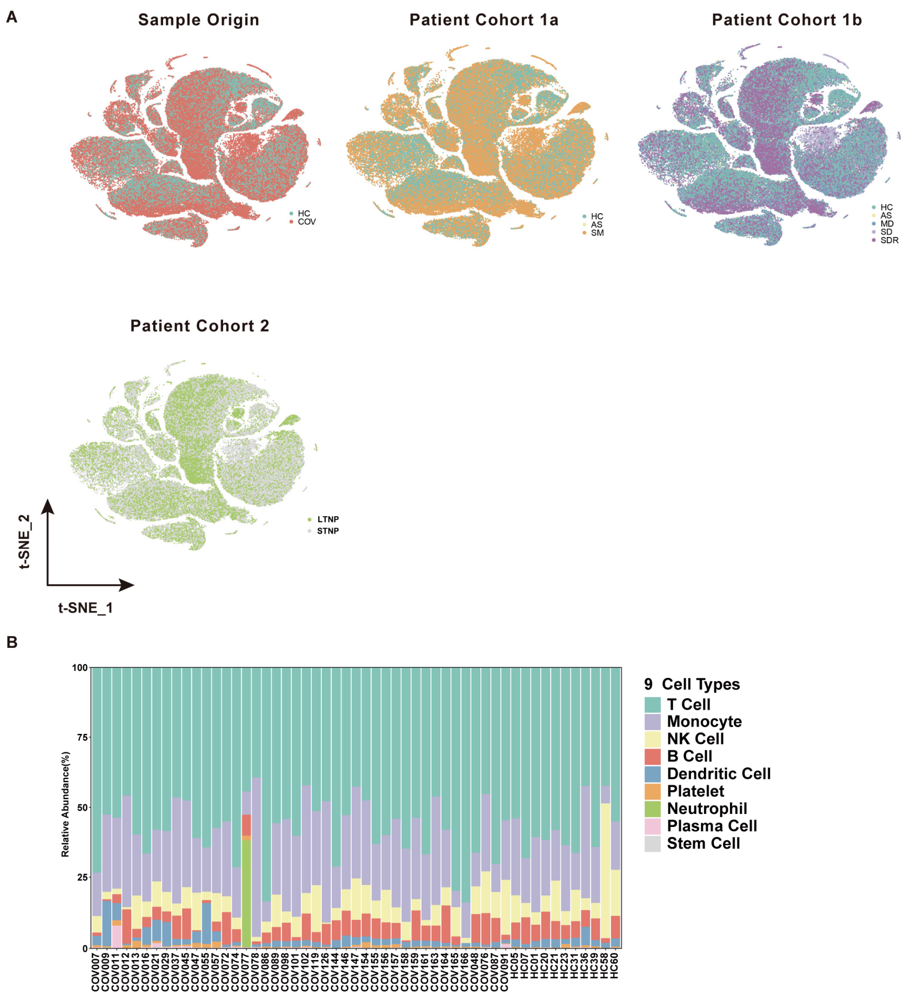

Fig. S1. Identification of nine major cell types in the PBMCs by scRNA-seq (related to Fig. 1).

(A) The T-distributed stochastic neighbor embedding (t-SNE) plots of 119,799 cells collected from all participants and distribution of each cohort. **(B)** The stacked bar chart shows the proportion (relative abundance) of 9 cell types in all the samples examined, respectively.

Supplementary Figure 2

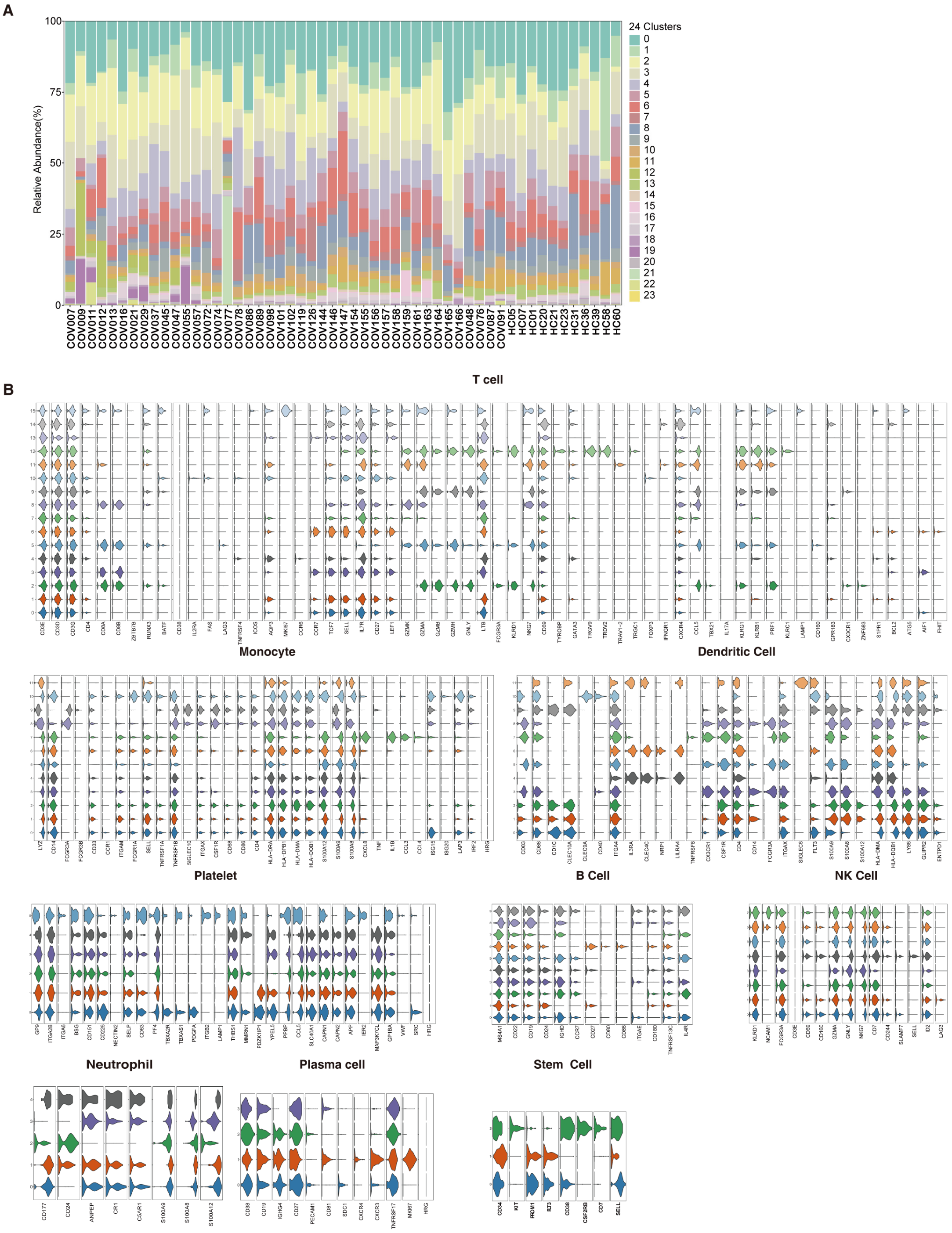

Fig. S2. Further identification of distinct cell clusters and sub-clusters in the PBMCs, (related to Fig. 2).

(A) The stacked bar chart shows the proportion (relative abundance) of 24 clusters in all the samples examined, respectively. **(B)** The violin plots showing the expression pattern of the selected cell markers in each of the 9 major cell types. (i.e., T cell, Monocyte, NK cell, B cell, Dendritic cell, platelet, neutrophil, plasma cell, and stem cell) identified from this study.

Supplementary Figure 3

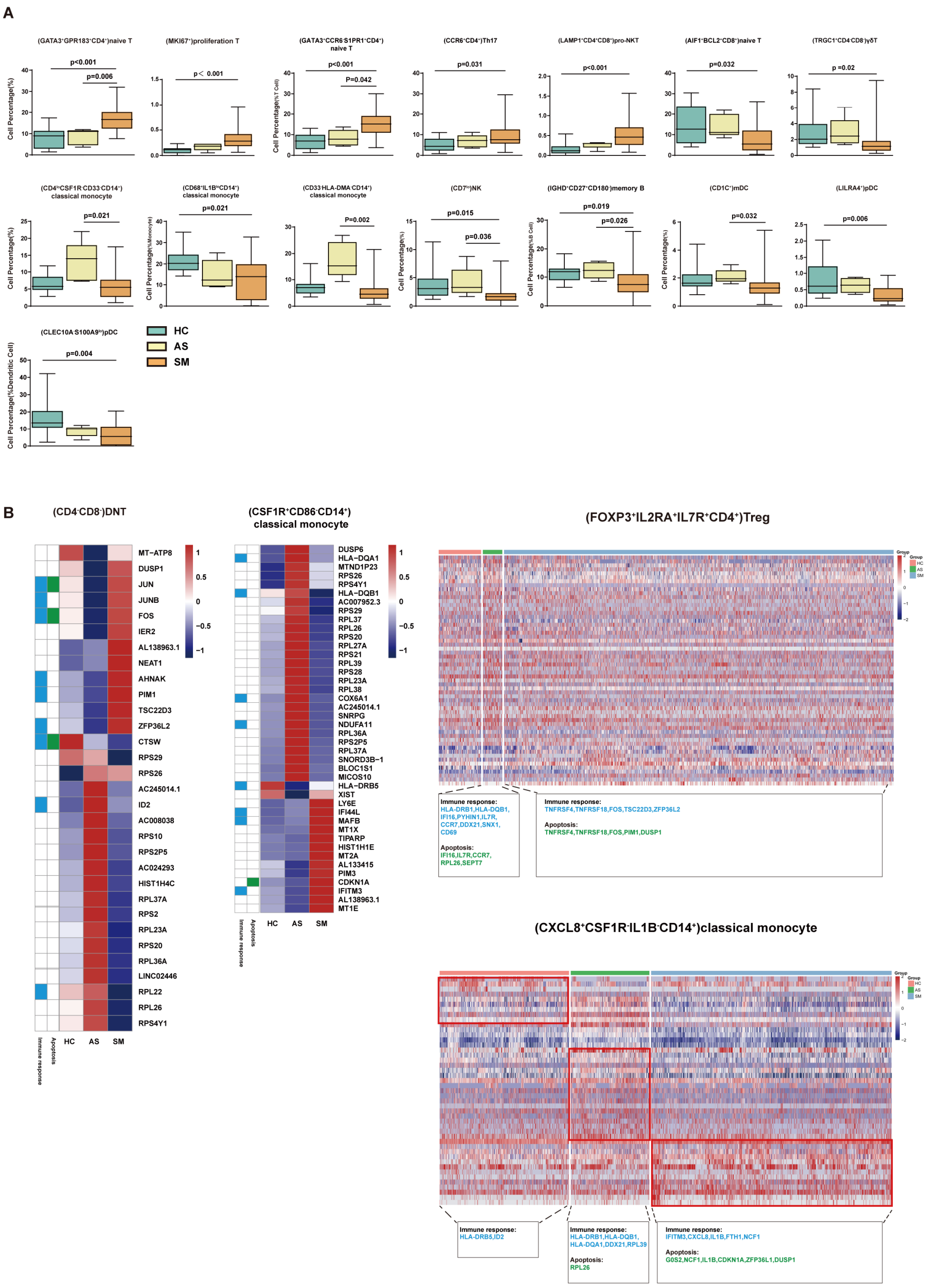

C

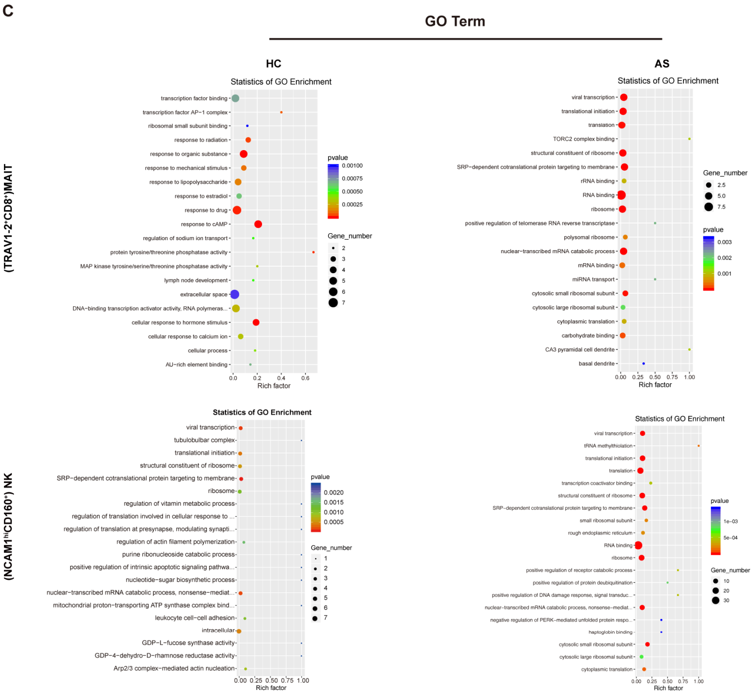
GO Term
HC
AS
Statistics of GO Enrichment
(TRAV1-2+CD8+)MAIT
(NCAM1hiCD160+) NK
pvalue
Gene_number
Rich factor

D

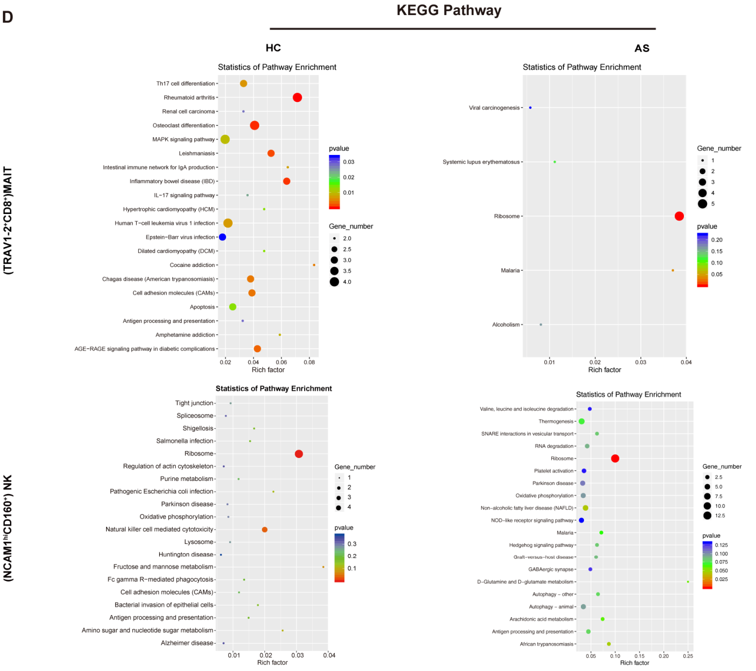
KEGG Pathway
HC
AS
Statistics of Pathway Enrichment
(TRAV1-2+CD8+)MAIT
(NCAM1hiCD160+) NK
pvalue
Gene_number
Rich factor

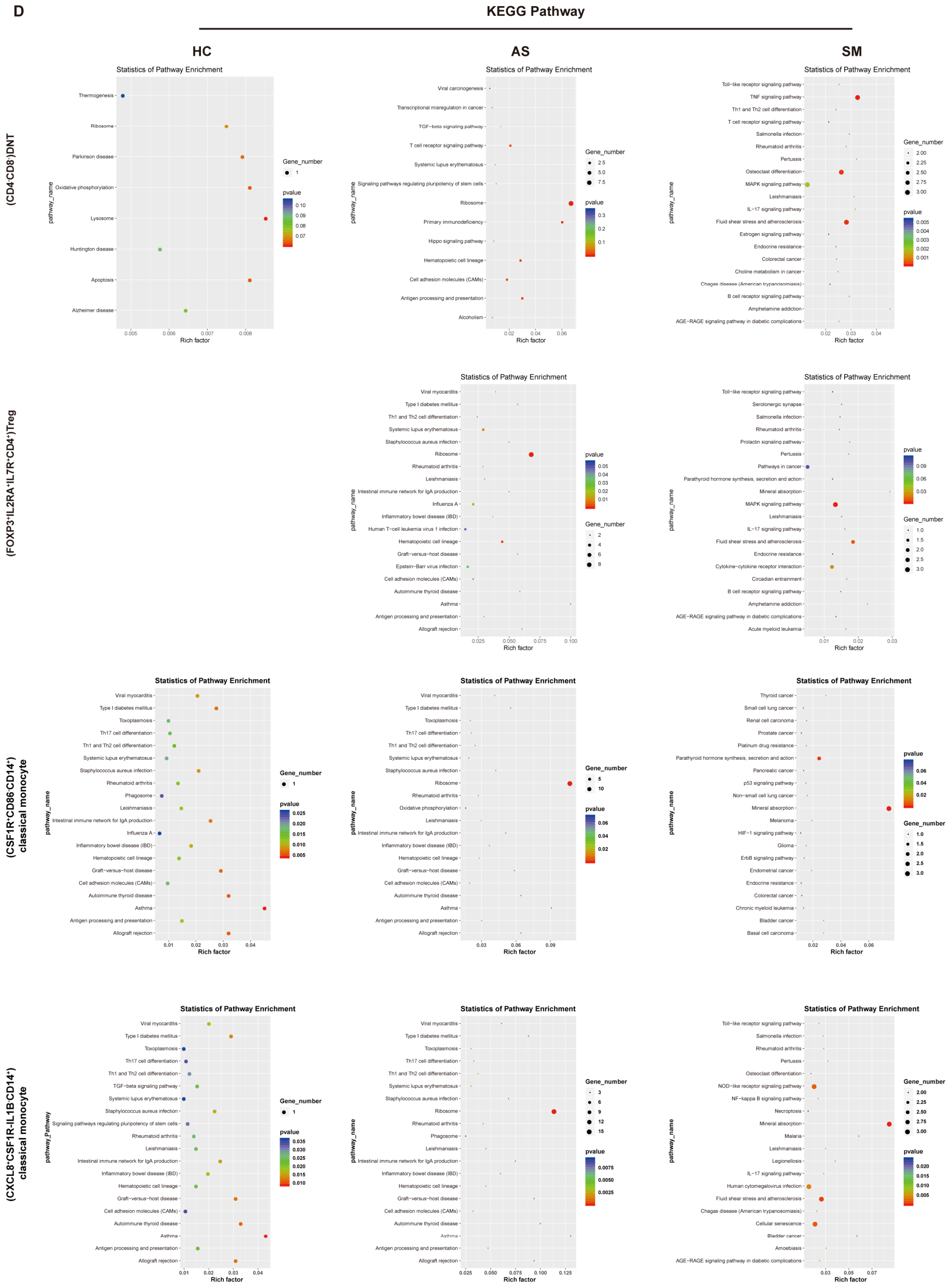
D
KEGG Pathway
HC
AS
SM
Statistics of Pathway Enrichment
(CD4⁻CD8⁻)DNT
(FOXP3⁺IL2RA⁺IL7R⁺CD4⁺)Treg
(CSF1R⁺CD86⁺CD14⁺) classical monocyte
(CXCL8⁺CSF1R⁺IL1B⁺CD14⁺) classical monocyte
pathway_name
Rich factor
Gene_number
pvalue

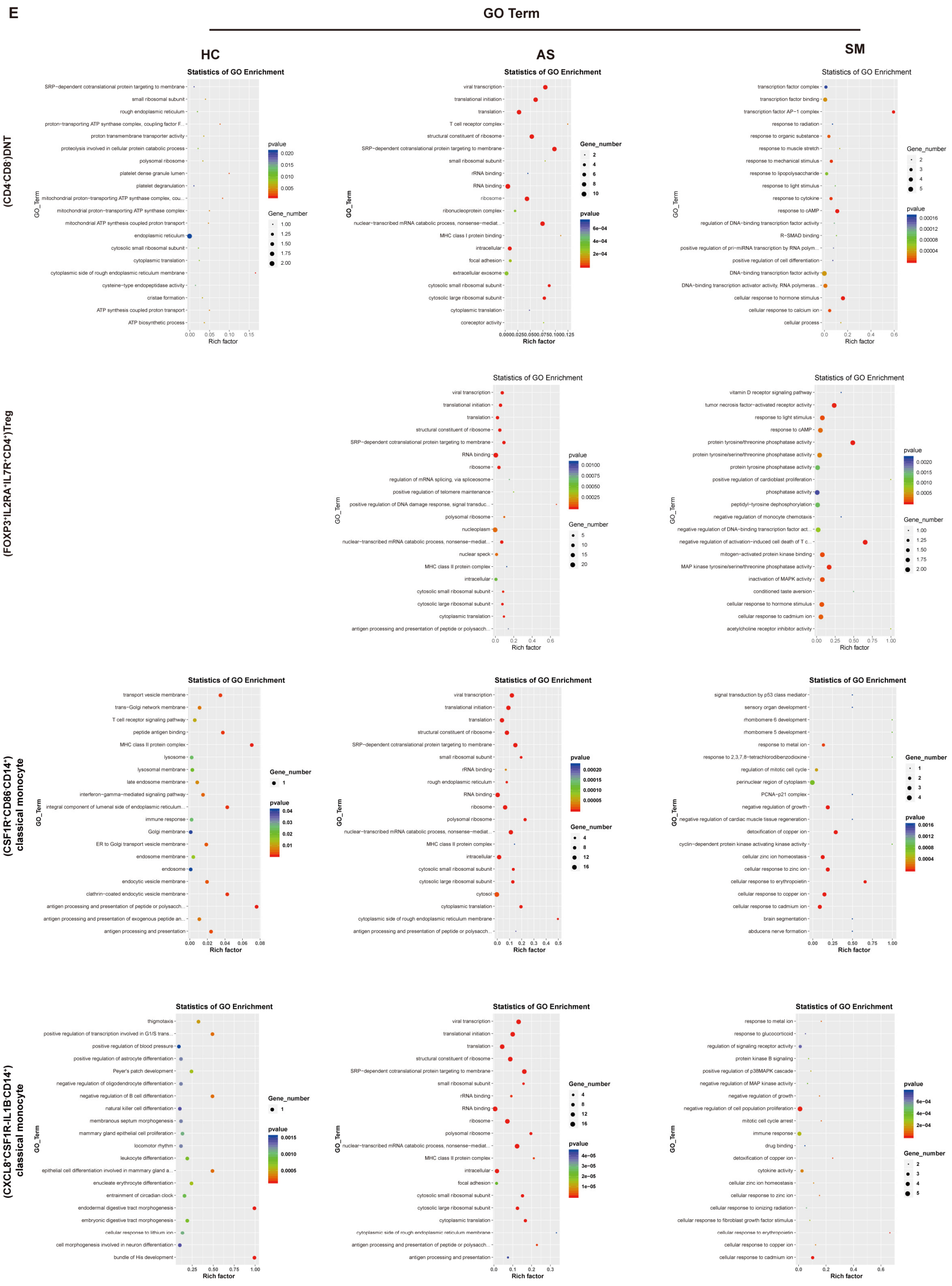
E
GO Term
HC
AS
SM
(CD4⁻CD8⁻)DNT
(FOXP3⁺IL2RA⁺IL7R⁻CD4⁺)Treg
(CSF1R⁺CD86⁺CD14⁺) classical monocyte
(CXCL8⁺CSF1R⁺IL1B⁺CD14⁺) classical monocyte
Statistics of GO Enrichment
Rich factor
GO_Term
pvalue
Gene_number

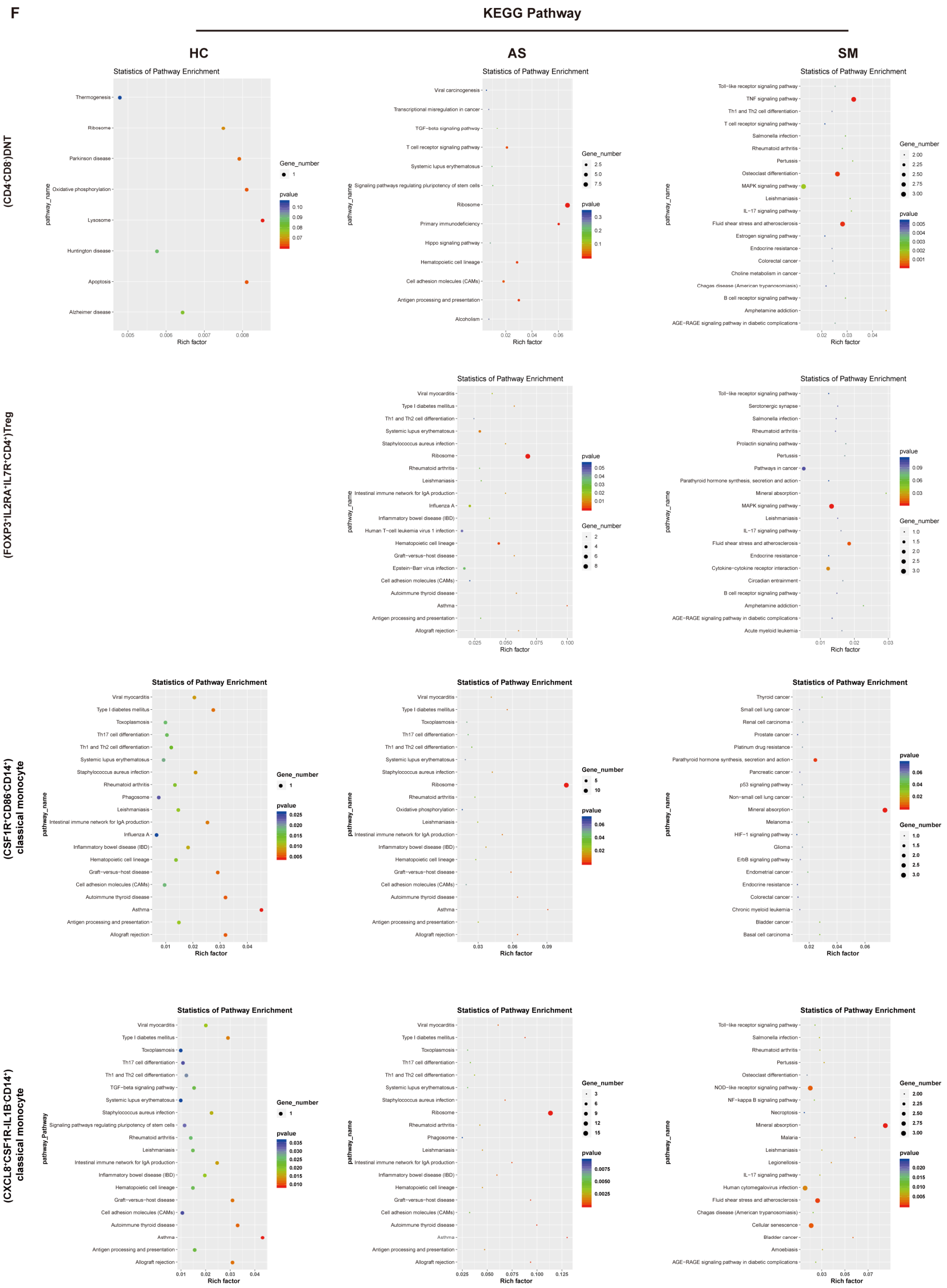
F
KEGG Pathway
HC
AS
SM
(CD4⁻CD8⁻)DNT
(FOXP3⁺IL2RA⁺IL7R⁺CD4⁺)Treg
(CSF1R⁺CD86⁺CD14⁺) classical monocyte
(CXCL8⁺CSF1R⁺IL1B⁺CD14⁺) classical monocyte
Statistics of Pathway Enrichment
Rich factor
pathway_name
Gene_number
pvalue

Fig. S3. Distinct immune cells associated with asymptomatic and symptomatic COVID-19 patients (related to Fig. 3 and Fig. 4).

(A) The Box and whisker plots showing the percentage of 15 cell subsets that had significant differences ($p < 0.05$) between the health control (HC), asymptomatic (AS), and symptomatic (SM) groups. The horizontal lines, box and whiskers correspond to median value, interquartile range (IQR) and minimum/maximum value, respectively. **(B)** The Heatmaps showing the differentially expressed genes (DEGs) with $p < 0.05$ and log2 fold change (FC) ≥0.26 in HC, AS and SM. The plots for ($CD4^-$ $CD8^-$)DNT cell and ($CSF1R^+CD86^-CD14^+$)classical monocyte represent the average expression level of each group. The plots for ($FOXP3^+IL2RA^+IL7R^+CD4^+$)Treg and ($CXCL8^+CSF1R^-IL1B^-$ $CD14^+$)classical monocyte show the expression of single cell from each group, which are centered and scaled via Pheatmap. Expression value of genes calculated via LogNormalize method of the "NormalizeData" function of the Seurat software. (**C**) Gene Ontology (GO) analysis. Top 20 significant GO terms sorted by −log10 (p value) from HC and AS were shown in (TRAV1-2^+CD8^+)MAIT and ($NCAM1^{hi}CD160^+$)NK cells, respectively. (**D**) KEGG pathway analysis. Top 20 significant KEGG pathways sorted by −log10 (p value) from HC and AS were shown in (TRAV1-2^+CD8^+)MAIT and ($NCAM1^{hi}CD160^+$)NK cells, respectively. **(E)** Gene Ontology (GO) analysis. The top 20 GO terms sorted by p value, gene number and rich factor from HC, AS, and SM. The absent plot (e.g. ($FOXP3^+IL2RA^+IL7R^+CD4^+$)Treg in HC group) indicates that there were no enriched GO terms in that group. **(F)** KEGG pathway analysis. The top 20 KEGG pathways sorted by p value, gene number and rich factor from HC, AS, and SM. The absent plot (e.g. ($FOXP3^+IL2RA^+IL7R^+CD4^+$)Treg in HC group) indicates that there were no enriched GO terms in that group.

Supplementary Figure 4

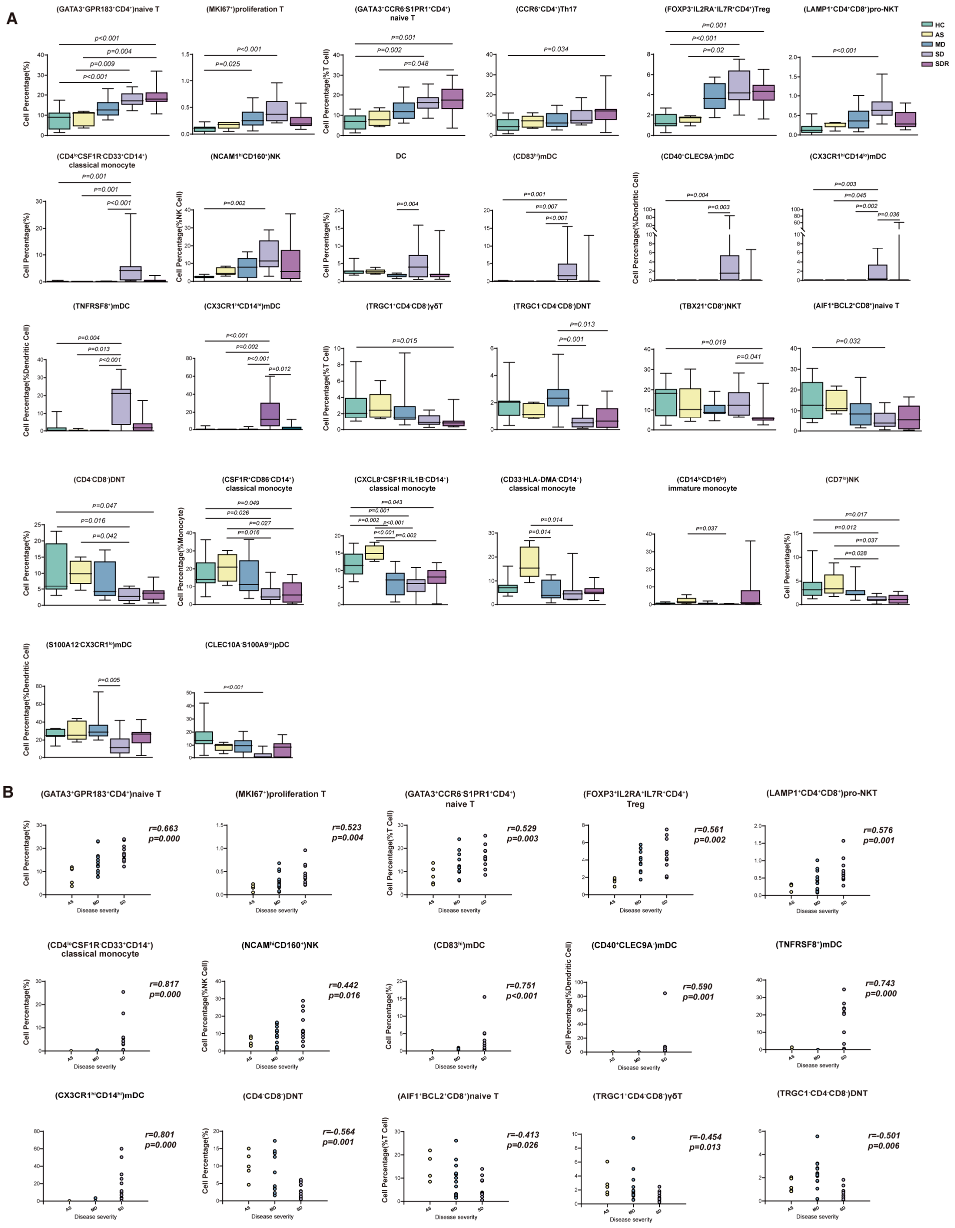

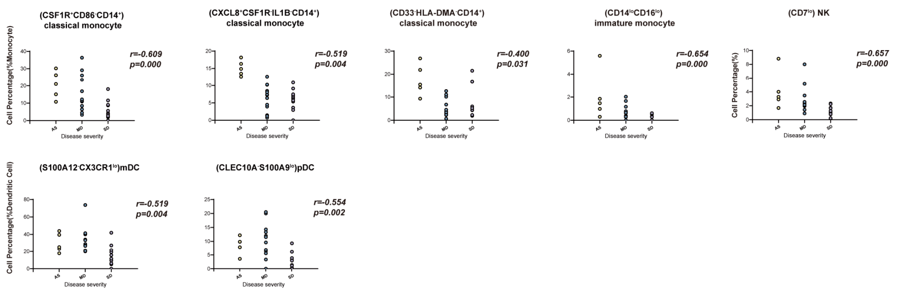

C

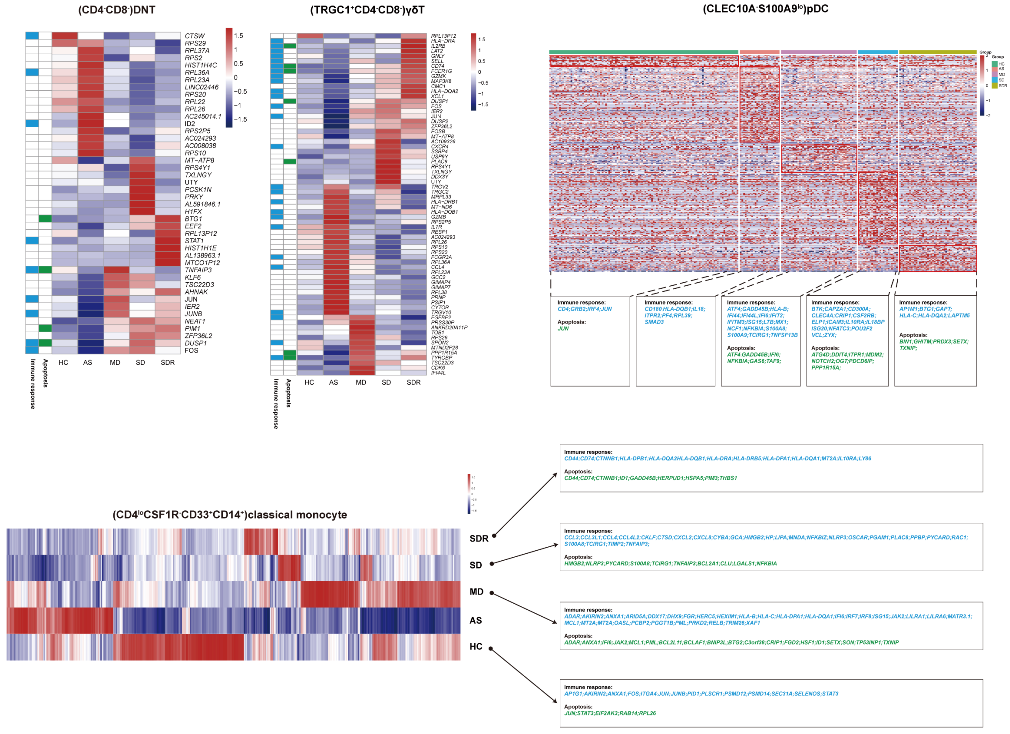

D

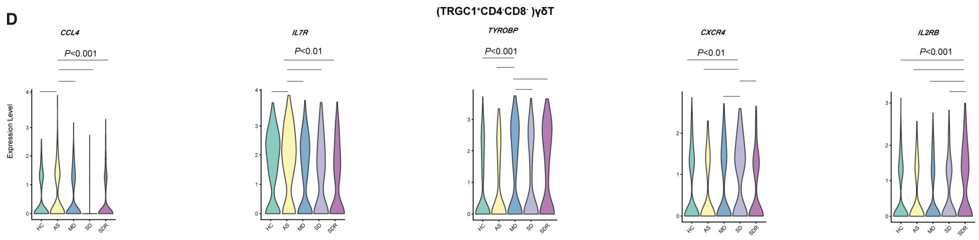

E

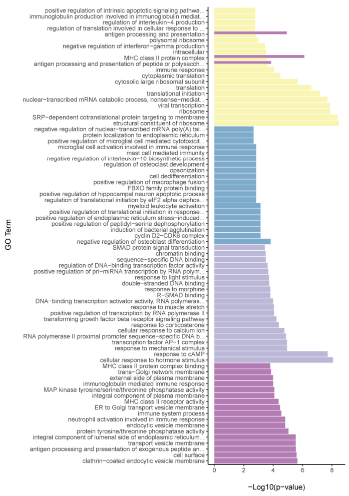

F

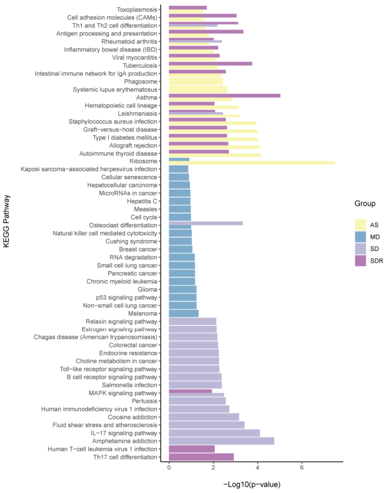

(TRGC1^{+}CD4^{-}CD8^{-})γδT

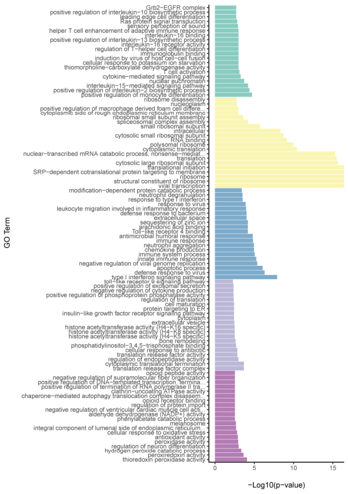

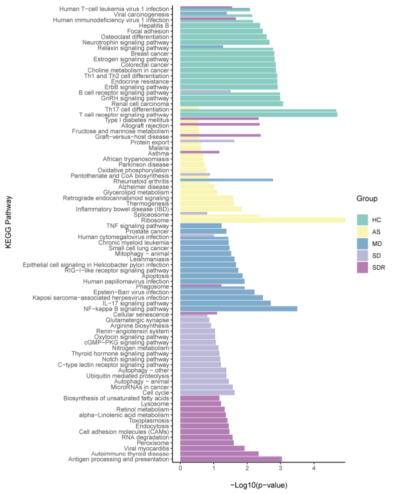

(CLEC10A^{-}S100A9^{lo})pDC

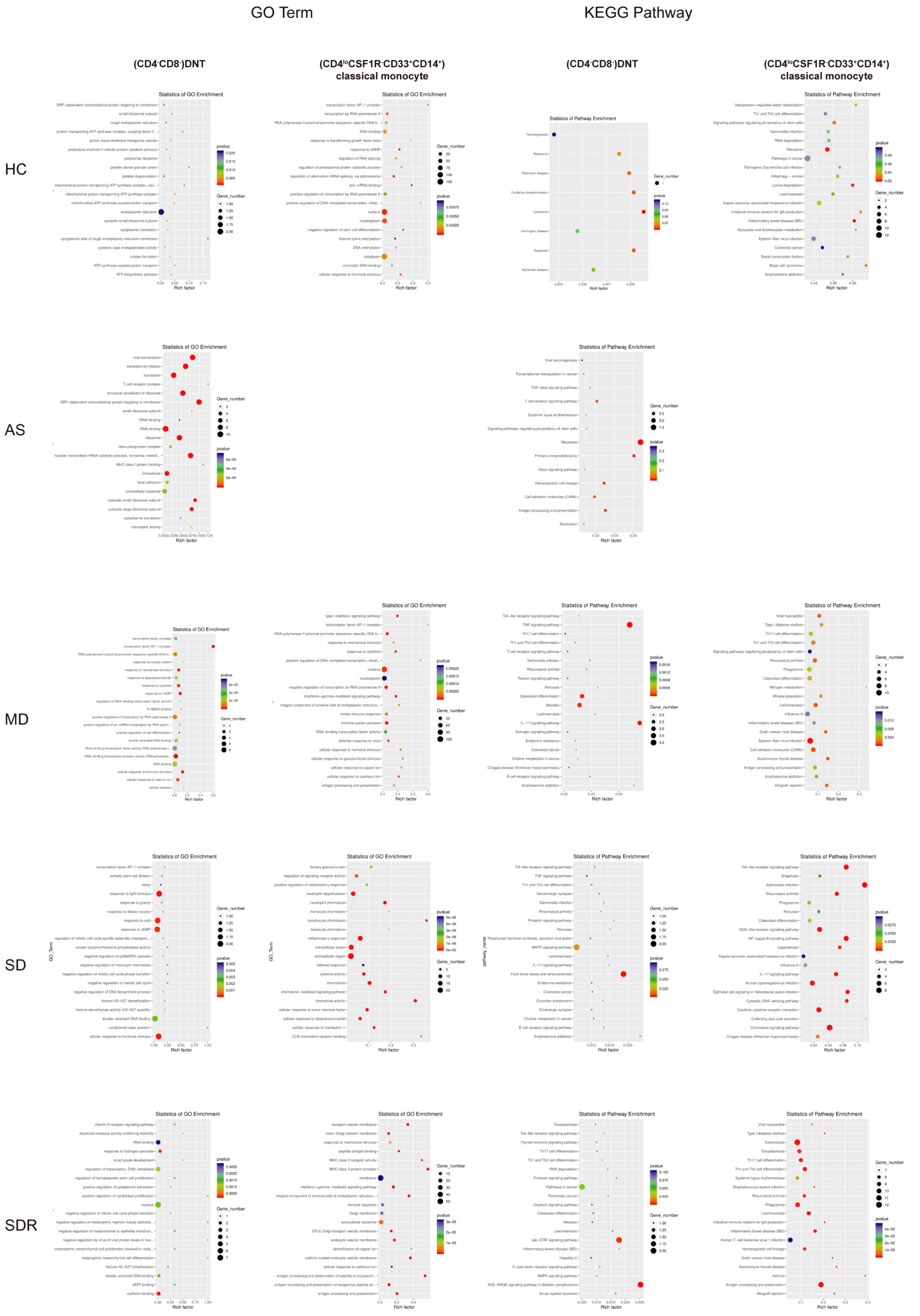
GO Term
KEGG Pathway
(CD4$^-$CD8$^-$)DNT
(CD4^{lo}CSF1R$^-$CD33$^+$CD14$^+$)
classical monocyte
(CD4$^-$CD8$^-$)DNT
(CD4^{lo}CSF1R$^-$CD33$^+$CD14$^+$)
classical monocyte
HC
AS
MD
SD
SDR
Statistics of GO Enrichment
Statistics of Pathway Enrichment

Fig. S4. Immune cell subsets associated with disease severity of COVID-19 patients (related to Fig. 5).

(A) The Box and whisker plots showing the percentage of 26 cell sets that had significant differences ($p < 0.05$) between the health control (HC), asymptomatic (AS), moderate disease (MD), severe disease (SD) and SD recovery patients (SDR) groups. The horizontal lines, box and whiskers correspond to median value, interquartile range (IQR) and minimum/maximum value, respectively. **(B)** Spearman rank order correlation analysis showing the significant association between the cell subset and the disease severity. **(C)** Detection of differentially expression genes (DEGs) with $p < 0.05$ and log2FC ≥ 0.26 between HC, AS, MD, SD and SDR in various cell subsets. The plots of ($CD4^-CD8^-$)DNT, ($TRGC1^+CD4^-CD8^-$)γδT and ($CD4^{lo}CSF1R^-CD33^+CD14^+$)classical monocytes represented the average expression of each group, whereas the ($CLEC10A^-S100A9^{lo}$)pDC plot showed the expression of single cell from each group. Expression value of genes calculated via LogNormalize method of the "NormalizeData" function of the Seurat software. Expression data in ($CLEC10A^-S100A9^{lo}$)pDC plot is centered and scaled on the row direction via Pheatmap. **(D)** The violin plot exhibits the expression of representative DEGs (*CCL4, IL7R, TYROBP, CXCR4,* and *IL2RB*) in ($TRGC1^+CD4^-CD8^-$)γδT cells, which were involved in apoptosis and/or immune responses. $p < 0.05$ are indicated. **(E)** Gene enrichment analyses of DEGs. The top 20 GO terms are labeled with name, and sorted by −log10 (p value) or p value, gene number and rich factor. The absent plot (e.g. ($CD4^{lo}CSF1R^-CD33^+CD14^+$) classical monocyte in AS group) indicates that there were no enriched GO terms in that group. **(F)** Gene enrichment analyses of DEGs. The top 20 KEGG pathways are labeled with name, and sorted by −log10 (p value) or p value, gene number and rich factor. The absent plot (e.g. ($CD4^{lo}CSF1R^-CD33^+CD14^+$) classical monocyte in AS group) indicates that there were no enriched KEGG pathways in that group.

Supplementary Figure 5

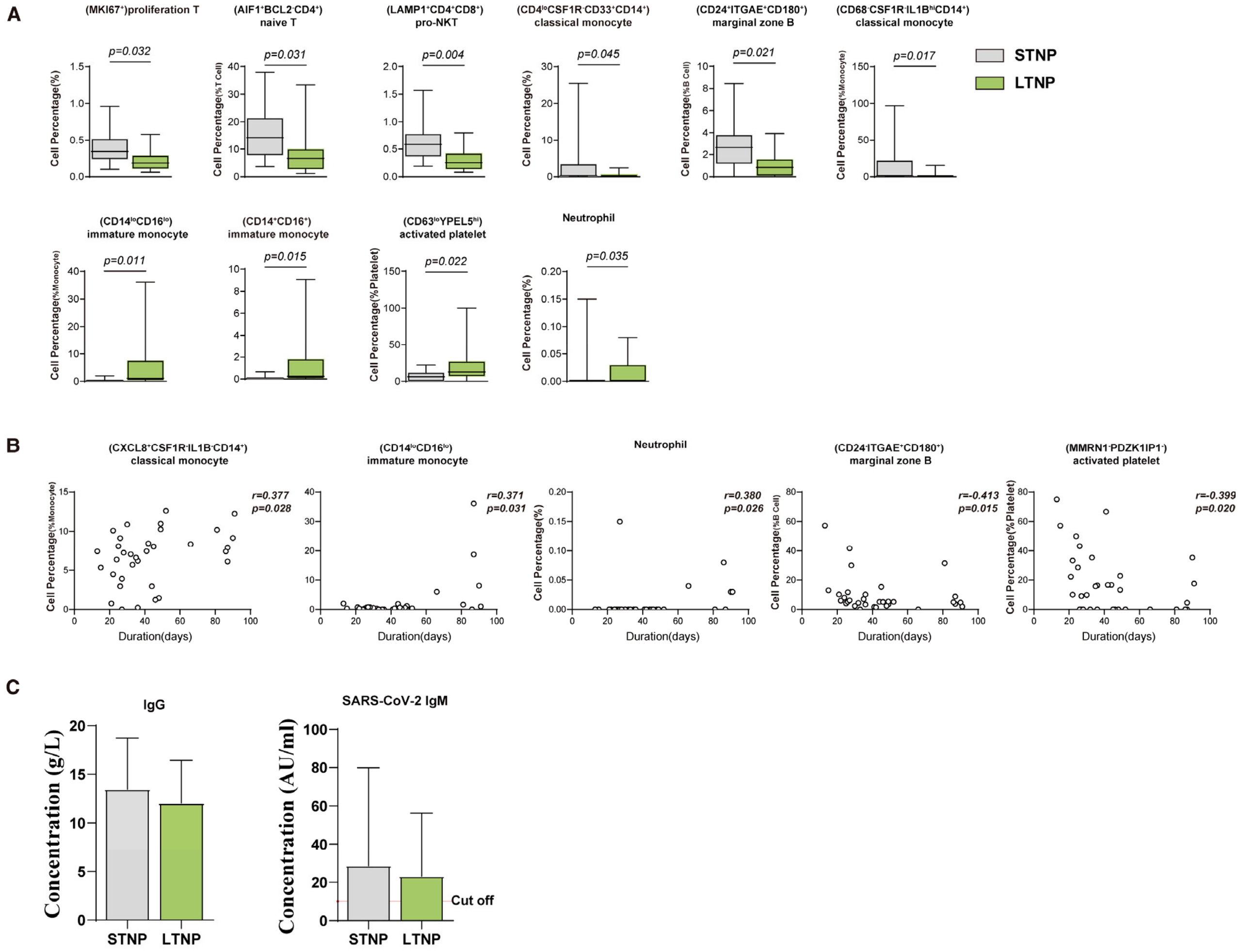

Fig. S5. Immune cell subsets associated with the patients of short-term nucleic acid test positive (STNP) and long-term nucleic acid test positive (LTNP) (related to Fig. 6).

(A) The Box and whisker plots showing the percentage of 10 cell sets that had significant differences ($p< 0.05$) between the STNP and LTNP groups, respectively. The horizontal lines, box and whiskers correspond to median value, interquartile range (IQR) and minimum/maximum value, respectively. **(B)** Spearman rank order correlation analysis showing the percentage of the immune cell subsets that are significantly associated with the STNP and LTNP, respectively. (C) The titer of antibodies (IgG and IgM) in the LTNP and STNP group obtained by routine Laboratory testing (Table S1). Error bars represent ± SD.
